# Recent Advances in Vision-Based Beef Cattle Body Measurement Technologies

**DOI:** 10.3390/ani16132058

**Published:** 2026-07-03

**Authors:** Xiaofan Deng, Fuli Zhang, Gang Jin, Liangyu Cui, Dongxu Zhang, Fa Zhang

**Affiliations:** 1 Tianjin Key Laboratory of High Performance Manufacturing Technology and Equipment, School of Mechanical Engineering, Tianjin University of Technology and Education, Tianjin 300222, China; dengxiaofan@tju.edu.cn (X.D.); 0321251011@tute.edu.cn (F.Z.); jgang1982@tute.edu.cn (G.J.); cuily@tju.edu.cn (L.C.); 2 School of Public Health, Xiamen University, Xiamen 361102, China; 3 College of Medical Technology, Beijing Institute of Technology, Beijing 100081, China

**Keywords:** body measurement, deep learning, non-contact measurement, point cloud, vision

## Abstract

Accurate beef cattle body measurement data are crucial for growth assessment, phenotypic analysis, breeding management, and precision livestock farming. Traditional manual measurements are labor-intensive, time-consuming, and likely to cause stress in animals, making it difficult to meet the demands of large-scale livestock farming. This paper employs a structured systematic literature review method, in accordance with the PRISMA 2020 guidelines, to summarize research progress in vision-based beef cattle body measurement. This paper focuses on reviewing technical approaches such as 2D image-based measurement, 3D measurement using RGB-D and LiDAR, and multi-view fusion. It analyzes key technologies including image segmentation, keypoint detection, point cloud processing, 3D reconstruction, and geometric calculations, and compares the advantages and disadvantages of different methods in terms of measurement accuracy, robustness, cost, and farm applicability. The results indicate that 2D image-based methods are low-cost and flexible to deploy but have limited expressiveness for 3D body measurement parameters; RGB-D and LiDAR methods can provide spatial information but are affected by point cloud noise, occlusion, equipment costs, and data processing complexity; multi-view fusion can improve the completeness of body surface information but places high demands on calibration, registration, and system integration. Current research still faces challenges such as a lack of public datasets, inconsistent annotation standards, uncertainty regarding ground truth, insufficient cross-ranch generalization validation, and limited practical applications. Future research should focus on developing standardized datasets, conducting cross-scenario validation, advancing multimodal perception, creating lightweight models, and applying edge computing to drive the evolution of visual body measurement toward continuous monitoring and intelligent decision-making.

## Simple Summary

Accurate beef cattle body measurements are important for growth monitoring, breeding, and precision livestock farming. Traditional manual measurements are labor-intensive and may stress animals. Vision-based technologies provide a non-contact and efficient alternative. This review summarizes recent advances in camera- and sensor-based beef cattle body measurement methods, compares their advantages and limitations, and discusses current challenges. Future research should focus on improving data quality, model robustness, and practical farm applications to support intelligent livestock management.

## 1. Introduction

Beef cattle production is shifting from experience-based management to data-driven management, with improving production efficiency [1], ensuring animal welfare [2], and achieving sustainable development being important goals of this transition [3]. Body measurements are fundamental for evaluating the growth of cattle [4], breed selection [5], reproductive performance [6], and health monitoring [7]. In the context of precision livestock farming, computer vision can not only replace some manual observation and measurement but also support downstream applications such as individual identification, live weight estimation, body condition scoring (BCS), and health monitoring. It should be noted that body measurement, live weight estimation, and body condition scoring represent distinct research objectives: body measurement aims to obtain morphological parameters such as height, length, and chest circumference; live weight estimation focuses on predicting individual weight; and body condition scoring is used to evaluate an animal’s energy reserves and nutritional status. Although these three tasks often share visual processing techniques such as image segmentation, keypoint detection, and 3D reconstruction, their output metrics and evaluation methods differ. Gilbert et al. [8] analyzed the effects of long-term selection on beef cattle body structure by measuring indicators such as hip height, withers height, body length, and chest girth of Angus and Hereford beef cattle. Bozkurt et al. [9] used indicators such as chest girth, withers height, body length, body depth, and hip width to predict the body weight of Brown Swiss cattle. Ozkaya and Bozkurt et al. [10] further compared the relationships between multiple body measurement indicators and body weight in different types of beef cattle, pointing out that chest girth is a relatively effective parameter for predicting body weight. Overall, indicators such as withers height, body length, chest girth, abdominal girth, and hip width can reflect skeletal development, body structure, and growth status of individuals, and are closely related to live weight, reproductive performance, production performance, and body condition assessment [11,12]. In the context of large-scale farming and the rapid development of precision livestock farming, continuously obtaining accurate, low-interference individual body measurement data has become an important requirement for beef cattle phenotype information collection and intelligent management.

Traditional body measurement primarily relies on manual contact operations, usually performed by trained personnel using a measuring stick, tape measure, body measurement device, or weighing equipment to measure beef cattle. Early research has used manually measured indicators such as chest girth, body length, withers height, and hip width to predict beef cattle weight and evaluate body conformation. Although these methods are intuitive to operate and the equipment cost is relatively low, they have obvious limitations in practical production [13,14]. First, manual measurement usually requires driving the beef cattle into a measuring pen or restraint device and requires the animals to maintain a relatively stable posture during measurement. This not only increases operation time and labor intensity but may also cause stress responses in the animals, affecting animal welfare and measurement posture [15]. Second, measuring indicators such as body height, body length, chest girth, and abdominal girth depends on the operator’s identification and localization of anatomical measurement points. The measurement results are easily affected by the operator’s experience, operational habits, and reading errors, leading to insufficient data repeatability and comparability [16]. In addition, in large-scale farming scenarios, manually measuring each animal is difficult to meet the demands for large populations, high frequency, and continuous monitoring, and close contact with large animals also increases safety risks for operators. Therefore, although traditional manual measurement methods are still an important basis for obtaining body measurement data, their labor-intensive, time-consuming, subjective, and highly intrusive characteristics limit their long-term application in precision livestock farming.

Addressing the shortcomings of traditional methods, computer vision technology provides continuous, remote, and non-contact solutions for measuring beef cattle body size [17]. This technology obtains images, depth maps, or 3D point clouds of beef cattle through devices such as RGB cameras, RGB-D sensors, stereo cameras, or LiDAR, and combines methods like object detection, image segmentation, keypoint localization, 3D reconstruction, and geometric computation to achieve automatic extraction of body size parameters. Compared with manual measurements, visual measurement can reduce interference with the natural behavior of animals, minimize errors caused by human contact and subjective judgment, and also has advantages such as data preservation, process traceability, and suitability for continuous monitoring [18,19]. In the context of precision livestock farming, computer vision can not only replace some manual observation and hand measurements but also be integrated with tasks such as individual identification, weight estimation, body condition scoring, and health monitoring, providing more objective and real-time data support for individualized management of beef cattle and group production decision-making.

**Figure 1:**
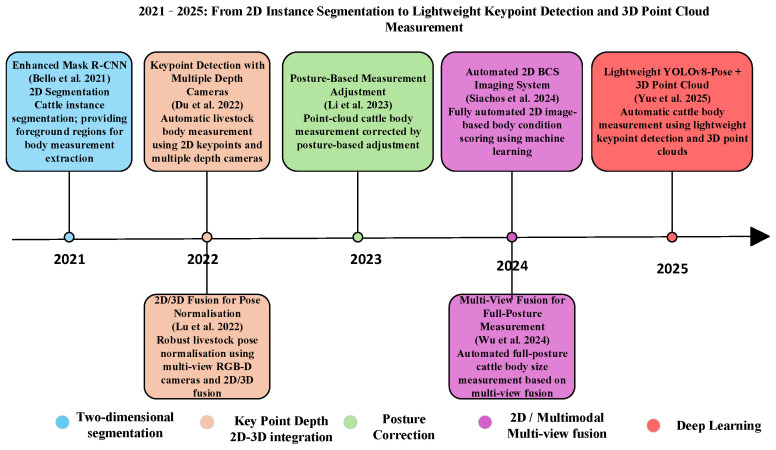
The development and evolution of beef cattle body measurement technology in the past five years [23,24,25,26,27,28,29].

In recent years, with the development of computer vision, deep learning, and 3D reconstruction technologies, vision-based beef cattle body measurement methods have gradually evolved from traditional 2D image analysis to RGB-D perception, multi-view fusion, and 3D point cloud intelligent measurement [1]. Significant progress has been made in areas such as keypoint detection, point cloud reconstruction, and lightweight network deployment, as shown in Figure 1. Different methods have their own characteristics in terms of hardware cost, spatial information acquisition capability, measurement accuracy, and environmental adaptability. 2D image methods are simple to deploy and low-cost but have limited capability in expressing 3D parameters such as chest girth, body depth, and body width; RGB-D and LiDAR methods can provide richer spatial information but are more susceptible to point cloud noise, occlusion, posture variations, equipment costs, and data processing complexity [20]; multi-view fusion systems can improve the problem of information loss from a single viewpoint but impose higher requirements on sensor calibration, point cloud registration, and system maintenance [21]. Therefore, when evaluating vision-based body measurement methods, it is necessary not only to compare individual measurement errors but also to consider acquisition conditions, algorithm processes, system complexity, and farm application scenarios [22].

Existing research has proposed various technical approaches for the automated measurement of beef cattle body dimensions; however, overall, these studies tend to be conducted separately around different objectives, resulting in a noticeable lack of integration. Some studies focus on live weight estimation, body condition scoring, or the prediction of individual body dimension parameters, leaning more toward modeling for specific application tasks; others concentrate on visual processing tasks such as object segmentation, keypoint detection, depth estimation, or point cloud reconstruction, but typically address only a single stage of the measurement process. In contrast, systematic reviews of the complete task chain for visual body measurement remain rare, particularly in terms of cross-comparisons between different technical approaches; existing work often lacks a unified analytical framework. From a practical application perspective, the stability of body measurement depends not only on model architecture or algorithm design but is also influenced by a variety of factors, including acquisition methods, sensor types, imaging angles, animal posture, occlusion, and ambient lighting. These factors not only affect image quality but may also lead to unstable segmentation boundaries, inaccurate localization of anatomical landmarks, and incomplete 3D reconstruction, thereby reducing measurement accuracy. Without a comprehensive comparison of these aspects, it is difficult to determine whether a particular method is truly suitable for application in large-scale cattle farms. Therefore, it is necessary to systematically summarize research on vision-based body measurement of beef cattle from the perspectives of both technical approaches and measurement workflows, and to further analyze the advantages and limitations of different methods in actual production environments.

**Figure 2:**
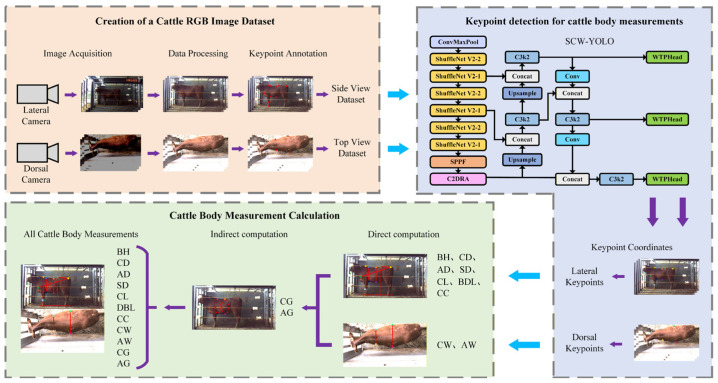
Body measurement process of beef cattle based on vision. (Reprinted from Reference [30], under the CC BY 4.0 license.) BH denotes body height; CD denotes chest depth; AD denotes abdominal depth; CW denotes chest width; AW denotes abdominal width; SH denotes sacral height; CL denotes croup length; DBL denotes diagonal body length; CC denotes cannon circumference; CG denotes chest girth; and AG denotes abdominal girth.

In light of the above issues, this paper employs a structured systematic literature review method to systematically summarize recent research on vision-based cattle body measurement, focusing on answering the following three questions: (1) What are the main technical approaches in vision-based beef cattle body measurement, and what are their core sensors, algorithmic workflows, and applicable measurement metrics, respectively? (2) What differences exist among methods such as 2D images, RGB-D, LiDAR, and multi-view fusion in terms of measurement accuracy, cost, robustness, data processing complexity, and suitability for ranch environments? (3) What methodological and practical limitations still exist in existing research regarding dataset construction, ground truth acquisition, evaluation metrics, cross-scenario generalization, and actual production deployment? To address these research questions, this paper developed a literature search and screening process in accordance with the PRISMA 2020 guidelines and established an analytical framework covering data acquisition, object segmentation, keypoint detection, 3D reconstruction, point cloud processing, and body measurement parameter calculation, as illustrated in Figure 2 [30]. Building on this foundation, this paper compares the advantages and limitations of different technical approaches, summarizes the major challenges facing automatic measurement systems in complex pasture environments, and further discusses future development directions such as standardized datasets, cross-pasture generalization validation, multi-source sensor fusion, real-time visual phenotyping, and digital twins for livestock, with the aim of providing guidance for the evolution of visual beef cattle body measurement technology from single-measurement to continuous monitoring and intelligent decision-making [31].

## 2. Literature Search Strategies, Screening Processes, and Methodological Frameworks for Systematic Reviews

This paper employs a structured systematic literature review (SLR) method to systematically organize and analyze vision-based body measurement technologies for beef cattle. The research methodology adheres to the PRISMA 2020 (Preferred Reporting Items for Systematic Reviews and Meta-Analyses) guidelines to ensure transparency, reproducibility, and methodological rigor in the literature screening process. This study did not conduct a quantitative meta-analysis but primarily employed a qualitative synthesis approach to compare and summarize different technical approaches [32].

To enhance the clarity of the methodological structure, this paper divides the entire review process into three consecutive stages: literature search strategy and inclusion/exclusion criteria; data extraction and structured coding; and synthesis and analysis of results. This process runs throughout the analytical framework of the paper and serves as the basis for comparing different visual body measurement methods.

### 2.1. Literature Search Strategy and Inclusion and Exclusion Criteria

To systematically review research progress on visual-based body measurement of beef cattle, this study employed a structured literature search and a phased screening method for literature collection. The databases searched included Web of Science, Scopus, PubMed, IEEE Xplore, ScienceDirect, SpringerLink, and Google Scholar. All database searches were completed on 1 December 2025. No chemicals, reagents, commercial cell lines, commercial samples, or commercial biological materials were used in this review. These databases were selected primarily because they cover relevant fields such as agricultural science, animal science, computer vision, sensor technology, artificial intelligence, and engineering applications, thereby enabling a relatively comprehensive collection of research findings on beef cattle body measurements, visual phenotyping, and 3D perception. In addition to database searches, this study also conducted a retrospective review of the reference lists in the included literature and identified potentially overlooked representative studies through citation mining and manual supplementary searches.

Prior to the formal literature search, preliminary searches were conducted using several core keywords to assess the suitability of different keyword combinations and the relevance of the retrieved records. Based on the results, search terms were grouped into three categories: research subjects, measurement tasks, and visual technologies. Keywords for research subjects included “cattle”, “beef cattle”, “dairy cattle” and “livestock”. Keywords for measurement tasks included “body measurement”, “body size”, “body dimension”, “morphometric measurement”, “live weight estimation”, “body condition scoring” and “livestock phenotyping”. Keywords for visual technologies included “computer vision”, “machine vision”, “RGB image”, “RGB-D camera”, “depth camera”, “stereo vision”, “LiDAR”, “point cloud”, “3D reconstruction”, “multi-view fusion”, “keypoint detection”, “image segmentation” and “deep learning”. These keywords were combined using Boolean operators, and the basic search query was formulated as follows: (“cattle” OR “beef cattle” OR “dairy cattle” OR “livestock”) AND (“body measurement” OR “body size” OR “body dimension” OR “morphometric measurement” OR “live weight estimation” OR “body condition scoring” OR “livestock phenotyping”) AND (“computer vision” OR “machine vision” OR “RGB image” OR “RGB-D camera” OR “depth camera” OR “stereo vision” OR “LiDAR” OR “point cloud” OR “3D reconstruction” OR “multi-view fusion” OR “keypoint detection” OR “image segmentation” OR “deep learning”).

Given the differences among various databases in terms of subject headings, field settings, and search syntax, the search queries were appropriately adjusted during the actual search process. For Web of Science and Scopus, combined searches were primarily conducted in the title, abstract, and keyword fields; PubMed focused on studies related to animal phenotypes, body condition scoring, and biometrics; IEEE Xplore was primarily used to supplement engineering and technical literature on computer vision, deep learning, sensors, and 3D reconstruction; ScienceDirect, SpringerLink, and Google Scholar were used to broaden the search scope and supplement interdisciplinary research. The primary search period for this study was set from January 2020 to January 2026 to highlight recent developments in deep learning, RGB-D perception, LiDAR point clouds, and multi-view fusion technologies in the field of bovine body measurement. Representative early studies that clarify the origins of the technology, the principles of manual measurement, early vision-based measurement methods, and the evolution of key algorithms, and that are closely related to the analytical framework of this review, were identified through citation tracking. Therefore, the search timeframe was primarily intended to capture recent research progress rather than to exclude classic foundational studies. Therefore, the search timeframe is primarily used to delineate recent research progress, rather than as an absolute criterion for excluding classic foundational literature. This paper systematically reviews the technological evolution of the literature, covering topics such as 2D image measurement, RGB-D and LiDAR measurement, multi-view fusion, object segmentation, keypoint detection, and point cloud processing.

The inclusion criteria for the literature are as follows: First, the study subjects must be beef cattle; or, if the study subjects are other livestock, the visual measurement methods, sensor configurations, or morphological parameter extraction processes must have direct reference value for measuring beef cattle body dimensions. Second, the study covers body measurements such as height at withers, body length, chest width, chest depth, abdominal width, rump width, chest circumference, and abdominal circumference, or addresses topics closely related to body measurement, such as live weight estimation, body condition scoring, and visual phenotyping, Third, the study employs visual perception technologies such as RGB cameras, RGB-D cameras, stereo cameras, depth sensors, LiDAR, 3D point clouds, or multi-view fusion. Fourth, the literature provides relatively clear descriptions of data acquisition methods, sensor configurations, algorithmic workflows, experimental validation methods, or quantitative performance metrics. Fifth, the literature consists of formally published peer-reviewed articles in Chinese or English, and the full text is available to the extent necessary to assess the study’s content.

The exclusion criteria for the literature are as follows: First, studies that deal solely with disease detection, behavioral recognition, estrus monitoring, feeding behavior, or individual identification, and do not include analyses of body measurements, body conformation, body weight, or morphological parameters. Second, studies that merely introduce object detection, image classification, or sensor devices, but do not explain their connection to beef cattle body measurement or visual phenotyping. Third, studies lacking necessary research methods, data sources, sensor configurations, experimental validation, or quantitative metrics, making it impossible to assess their technical approach and measurement performance. Fourth, conference abstracts, editorial commentaries, news reports, patents, thesis abstracts, or other non-peer-reviewed materials. Fifth, duplicated studies, literature for which the full text is unavailable, and literature not published in Chinese or English.

**Figure 3:**
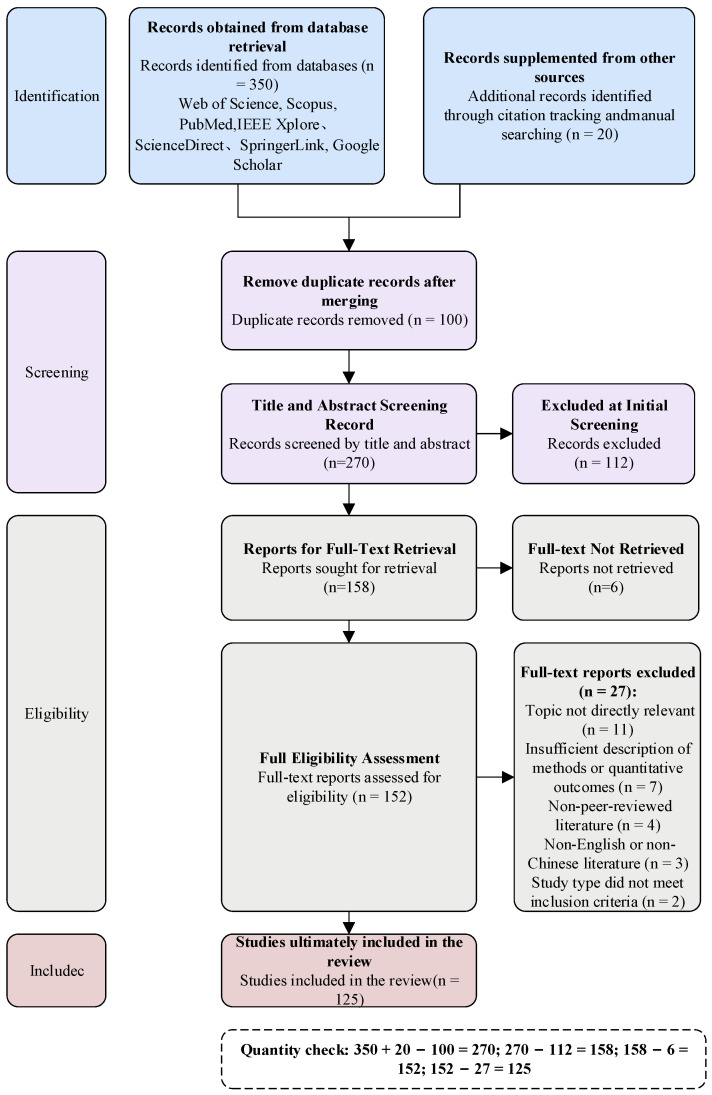
Search flow used in the review.

The literature screening process followed the PRISMA 2020 workflow. First, a total of 350 records were retrieved from the aforementioned databases, and an additional 20 records were obtained through reference tracing, citation mining, and manual searches. After merging the search results, 100 duplicate records were removed, leaving 270 publications that proceeded to the title and abstract screening stage. During the initial screening stage, the relevance of the literature was primarily assessed based on the study subjects, measurement tasks, and types of visual technology. A total of 112 records not directly related to the research topic were excluded, leaving 158 articles to proceed to the full-text retrieval stage. During full-text retrieval, 6 articles were excluded from eligibility assessment due to the unavailability of the full text; ultimately, 152 articles underwent full-text review. Based on the predefined inclusion and exclusion criteria, a total of 27 full-text articles were excluded. Among these, 11 were excluded because their research topics were not directly related to bovine body measurements; 7 were excluded due to insufficient methodological descriptions or quantitative indicators; 4 were non-peer-reviewed materials; 3 were not in Chinese or English; and 2 did not meet the inclusion criteria for study type. A total of 125 articles were ultimately included in the review analysis. The literature screening process strictly followed the PRISMA 2020 (Preferred Reporting Items for Systematic Reviews and Meta-Analyses) guidelines. The literature screening and exclusion procedures for each stage are detailed in the flowchart shown in Figure 3.

The ultimately included literature was categorized according to its primary technical approaches and research objectives. The technical approaches primarily included 2D RGB image measurement, RGB-D depth measurement, LiDAR point cloud measurement, 3D reconstruction, and multi-view fusion; the algorithmic aspects primarily included object detection and segmentation, keypoint localization, depth estimation, point cloud segmentation and registration, 3D model reconstruction, and body measurement parameter calculation. For studies involving multiple technical approaches, classification was based on their primary data sources, core measurement methods, and research contributions. Where necessary, these studies were discussed across different technical categories to avoid simplistic categorization based solely on sensor names or individual algorithms.

### 2.2. Data Extraction and Coding Methods

To ensure comparability among different studies, this paper conducted structured data extraction and organization for the 125 included studies following the literature screening process. This process was based on a pre-designed coding framework designed to transform highly heterogeneous studies into a unified analytical framework, thereby supporting subsequent quantitative and qualitative comparative analyses [33].

During the implementation process, data extraction was based on a unified field system, primarily covering basic research information, perception modalities, methodological workflows, anthropometric parameters, and evaluation metrics. Basic information, including publication year, research scenarios, and application objectives, was used to characterize the temporal evolution of technological development. Perception modalities were classified into RGB images, RGB-D data, LiDAR point clouds, and multi-view fusion systems to reflect application differences under various sensor conditions. Methodological workflows focused on documenting key stages such as object detection, semantic segmentation, keypoint localization, depth estimation, 3D reconstruction, and point cloud processing, thereby reconstructing the complete visual body measurement technology chain. Anthropometric parameters, such as height, length, chest circumference, and width, together with their calculation methods, were systematically organized to ensure consistency in measurement objectives across different studies. At the evaluation level, common metrics such as MAE, RMSE, MAPE, and R^2^ were extracted, and information on dataset size, experimental environments, and cross-scenario validation was supplemented wherever possible to assist in assessing model generalization capabilities.

Based on the above structured extraction results, this paper performs uniform coding and categorization of all literature. For studies involving multiple technical approaches, the primary category was determined based on the main data sources and core methods, with supplementary annotations added in other relevant categories to avoid information loss or oversimplification. The resulting coded database provides a data foundation for subsequent comparative analyses of different visual measurement methods in terms of spatial information acquisition capabilities, measurement accuracy, and engineering applicability. It also directly supports the statistics and summaries presented in the subsequent tables, thereby enhancing the traceability and consistency of the overall analytical process. Reference management and duplicate removal were performed using Zotero 7.0 (Corporation for Digital Scholarship, Vienna, VA, USA), and structured data extraction and coding were conducted using Microsoft Excel 2021 in Microsoft 365 (Microsoft Corporation, Redmond, WA, USA).

### 2.3. Sources of Error in Visual Body Measurement and an Evaluation Framework

In research on vision-based beef cattle body measurement, model performance is typically evaluated by comparing it to the ground truth. However, the methods used to obtain the ground truth vary across studies, and the ground truth itself is subject to a certain degree of uncertainty, which affects the comparability of results among different methods [17].

It should be further noted that in visual body measurement research, there is no single, unified “absolute truth” for ground truth. Existing studies typically use manual measurements, weighing systems, 3D scanning equipment, or expert annotations as reference standards; however, these methods themselves are subject to varying degrees of uncertainty and systematic errors. For example, manual measurements are significantly influenced by the operator’s experience and the definition of measurement points; weighing results reflect body mass rather than geometric body dimensions; although 3D scanning offers high accuracy, it remains subject to occlusion, pose, and registration errors; and expert annotations suffer from issues of subjective consistency. Therefore, the error metrics reported in different studies strictly depend on their respective reference truth systems, and results across studies are not fully comparable [20].

To systematically characterize the sources of error in visual body measurement, this paper constructs an error decomposition framework based on existing reviews of visual measurements in livestock and poultry and research on multisensor body measurement. It decomposes the overall measurement error into five major components: sensor and acquisition errors, algorithm and model errors, reference truth errors, animal-specific and scene errors, and evaluation and statistical errors [1,17].

Among these, sensor and acquisition errors primarily stem from camera calibration errors, depth and point cloud noise, limitations in resolution and ranging distance, and variations in lighting and occlusion conditions; algorithm and model errors mainly include deviations in object segmentation boundaries, keypoint localization errors, 3D reconstruction and registration errors, as well as geometric calculation and regression errors; reference truth errors stem from inter-observer variability, inconsistencies in the definition of measurement points, deviations in weighing or 3D scanning references, and the subjectivity of expert annotations; animal-specific and scene-related errors include changes in posture, motion blur, fur coverage and body surface deformation, as well as individual differences and environmental complexity; Evaluation and statistical errors are primarily manifested in insufficient sample size, dataset bias, inadequate cross-pasture generalization, and inconsistencies in evaluation metrics.

Therefore, the “overall error” in visual body measurement is not a reflection of a single model error, but rather the result of the coupling of errors from multiple sources. In particular, the uncertainty of the ground truth varies significantly across different studies, meaning that error metrics derived from different GT systems are not fully comparable in a strict sense. Therefore, when evaluating visual body measurement methods, both the source of the ground truth and its error range should be reported to enhance the interpretability and comparability of results across different studies.

## 3. Vision-Based Measurement Method for Beef Cattle Body Size

In visual body measurement research, the differences between methods mainly lie in the dimensions of spatial information acquisition and the capability to integrate data across perspectives. Accordingly, existing methods can be divided into three categories: two-dimensional visual measurement methods, three-dimensional reconstruction measurement methods, and multi-view fusion systems.

2D methods are mainly based on extracting contour, texture, or keypoint features from a single RGB image, and achieve body measurement parameter estimation through geometric modeling or statistical regression. Their characteristics are simple structure and low cost, but they lack depth information [34,35]. 3D methods, on the other hand, obtain spatial point cloud data through stereo vision, multi-view geometric reconstruction, structured light, or depth sensors, thereby achieving more accurate body modeling. However, 3D methods are usually sensitive to equipment costs, ambient lighting, and point cloud noise, and their data processing complexity is relatively high [1].

In recent years, multi-view fusion systems have gradually become a research hotspot. This type of method improves measurement stability and robustness by integrating image information from different viewpoints, without fully relying on high-cost depth sensors [36]. Multi-view fusion can serve both as an extension of two-dimensional methods and as a key step in three-dimensional reconstruction, offering a compromise between accuracy and system complexity. Therefore, it is necessary to systematically sort out and comparatively analyze the aforementioned three technical paths [21].

### 3.1. Method for Measuring Beef Cattle Body Dimensions Based on Two-Dimensional Image Features

Visual measurement systems based on two-dimensional RGB images were an important technical approach for early beef cattle body measurement. Due to their low cost, flexible deployment, and ease of implementation, they have received widespread attention in ranch settings. Such systems typically use one or more standard cameras to capture images of beef cattle from side, top-down, or rear views. They employ methods such as object detection, instance segmentation, keypoint localization, and regression modeling to extract body surface contours and morphological features, which are then used for body measurement parameter estimation. In some studies, the obtained body measurements or conformation features are also used as input for downstream tasks such as live weight estimation or body condition scoring; however, these tasks represent extended applications based on body measurement data, and their research objectives and evaluation metrics differ from those of body measurement itself. In recent years, with the development of deep learning, 2D RGB image methods have made significant progress in feature extraction automation, adaptability to complex backgrounds, and real-time deployment capabilities, with representative studies shown in Table 1.

Compared to 3D reconstruction or multisensor fusion approaches, 2D vision measurement systems offer advantages such as lower hardware costs and relatively simple data acquisition and processing workflows, making them more suitable for cost-sensitive applications, scenarios with limited deployment conditions, or those requiring moderate measurement accuracy [39]. As shown in Table 1, in recent years, 2D vision methods have gradually evolved from traditional measurement approaches relying on contours and manual features to automated measurement workflows that integrate object detection, image segmentation, keypoint localization, and deep learning-based regression. Such systems typically capture images of beef cattle from specific viewpoints using fixed cameras and extract body measurement parameters using geometric modeling or regression models [40]. Performance differences across studies are not only related to model architecture but are also closely linked to image acquisition conditions, sample size, annotation quality, animal posture, and the type of measurement metrics. Generally speaking, methods that incorporate keypoint detection or anatomical constraints perform more consistently for metrics with clear geometric definitions, such as body height, body length, and chest depth; whereas methods that rely directly on regression predictions based on overall image features, although simpler in process, are more susceptible to the effects of shooting angles, occlusion, background interference, and scale calibration errors.

**Figure 4:**
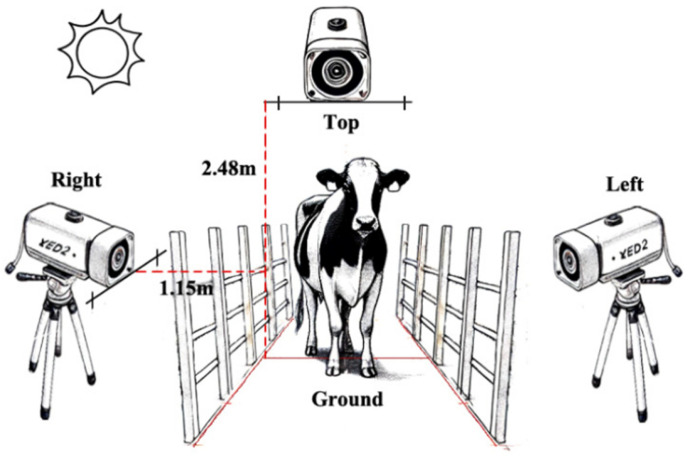
Deployment scheme of the two-dimensional vision measurement system [24].

A typical two-dimensional vision measurement system architecture is shown in Figure 4 [24]. Under natural outdoor lighting conditions, this system positions cameras above and on both sides of the aisle to capture image data of beef cattle from different viewpoints: the top camera is installed 2.48 m above the ground, directly above the aisle; the left and right cameras are positioned in the sunlit and shaded areas, respectively, and mounted on tripods 1.15 m from the aisle fence. Each Zed2 camera is connected to a Windows 11 host via USB 3.0, with image acquisition and processing handled by independent threads. This system demonstrates that two-dimensional vision methods offer good deployment flexibility in real-world ranch environments, but it also highlights their dependence on camera mounting positions, lighting conditions, and calibration accuracy. Since two-dimensional images inherently lack true depth information, such methods are feasible for measuring visible contour parameters such as body height and body length, but they still face accuracy limitations when measuring indicators with distinct three-dimensional structural features, such as chest circumference, abdominal circumference, body width, and chest depth. Therefore, when comparing the performance of 2D vision methods in future research, in addition to reporting error metrics, researchers should further specify the acquisition environment, posture control, calibration methods, and cross-scenario validation results to enhance comparability across studies and the practical reference value for real-world applications.

### 3.2. Measurement Method of Beef Cattle Body Size Based on 3D Reconstruction

In the field of three-dimensional visual body measurement, the key technology lies in how to reconstruct the three-dimensional surface structure and geometric information of the target object from two-dimensional image sources. Existing research can be mainly divided into two categories of 3D reconstruction measurement methods: systems based on RGB-D cameras and systems based on LiDAR. Both types of systems achieve 3D model construction by acquiring spatial depth information, but they differ significantly in data acquisition mechanisms, accuracy performance, cost, and environmental adaptability. Systems based on RGB-D cameras use compact depth sensors to capture color images and depth maps in real time and can directly output RGB-D data with depth information [41]; LiDAR systems obtain high-precision, high-density 3D spatial point cloud data by actively emitting laser pulses and measuring their return times, offering significant advantages in large-scale and complex environments [42]. Depending on the platform, LiDAR systems can be categorized into ground-based fixed LiDAR, mobile LiDAR, and UAV-mounted LiDAR. Among them, UAV-LiDAR has garnered widespread attention due to its high flexibility, broad coverage, and its ability to effectively avoid stress disturbance to animals. The following content will review the technical principles and application characteristics of these two types of 3D reconstruction methods respectively.

#### 3.2.1. RGB-D Based Method for Measuring Beef Cattle Body Size

The 3D body measurement system based on RGB-D cameras acquires RGB-D data with depth information by synchronously capturing color images and depth maps, making 3D spatial reconstruction real-time and cost-effective. In the scenario of livestock body measurement, existing studies have proposed various live weight estimation and shape reconstruction models based on RGB-D images. Among them, Ruchay et al. [41] used low-cost RGB-D sensors combined with deep learning regression models to predict and verify the live weight of beef cattle, demonstrating the potential of integrating RGB-D 3D reconstruction with regression. In addition, regarding the collection of depth image data for dairy and beef cattle, Kadlec et al. [43] achieved automatic depth map acquisition by installing RGB-D depth sensors above the beef cattle passage, providing an experimental basis for the extraction of body measurement parameters and 3D understanding from depth data. Ma et al. [44] systematically compared measurement methods based on RGB images, RGB-D sensors, and 3D point clouds, and analyzed the advantages and disadvantages of each data modality, illustrating the advantages of RGB-D devices in balancing real-time performance and cost-accuracy.

It is worth noting that the accuracy and applicability of RGB-D systems are constrained by the technical principles of depth sensors. Regarding changes in illumination, the measurement accuracy of depth cameras such as the Kinect v2 is significantly affected by ambient light intensity, and depth information is prone to failure under strong or low-light conditions; regarding surface reflection, noise generated by specular and diffuse reflections can interfere with the accurate acquisition of depth data; regarding texture features, cameras based on stereo vision principles have difficulty measuring distances on low-texture objects; regarding dynamic scenes, point cloud noise and motion blur caused by moving targets pose additional challenges to measurement accuracy. In addition, the effective depth measurement range of RGB-D cameras is usually 0.1 to 5.0 m, which is significantly limited in larger-scale or outdoor scenarios. Therefore, in practical deployment, it is necessary to select hardware solutions adapted to the specific characteristics of the scene, supplemented with targeted error compensation and data preprocessing strategies.

#### 3.2.2. Measurement Method of Beef Cattle Body Size Based on LiDAR

LiDAR acquires high-precision three-dimensional distance information by emitting laser beams and measuring the reflection time, generating dense and accurate point cloud data. Compared with RGB-D cameras, LiDAR is insensitive to changes in ambient lighting, has a longer measurement range, and higher accuracy, making it especially suitable for use in open pasture environments.

**Figure 5:**
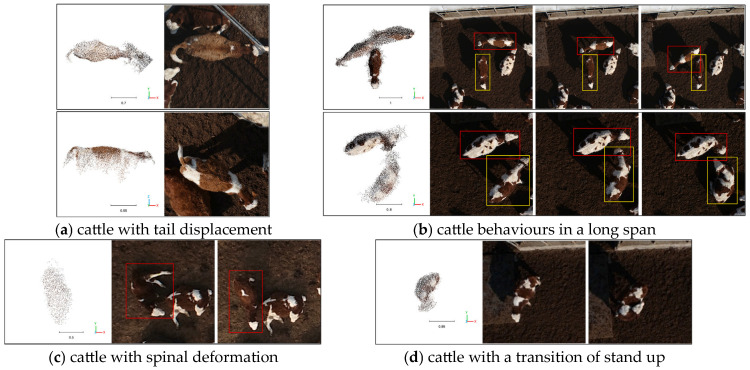
Schematic diagram of the 3D point cloud acquisition system for beef cattle based on LiDAR. (Reprinted from Reference [48], under the CC BY 4.0 license) (**a**) Example depicting tail movement. The top row shows the tail raised high with the body arched, while the bottom row shows a tail swinging action but with no body deformation. (**b**) The top row records stretching (red) and standing still (yellow), while the bottom row captures head-turning (red) and the transition from standing to lying down (yellow). (**c**) Imagining a beef cow turning its head and presenting a curved body posture. (**d**) Capturing the process of a beef cow standing up from a lying position.

In recent years, solid-state/semi-solid-state LiDAR has reduced or eliminated traditional mechanical rotating components in scanning mechanisms, promoting continuous optimization of sensors in terms of size, reliability, and large-scale cost, allowing them to gradually expand from automotive and robotic fields to agriculture and livestock scenarios that are more sensitive to cost and environmental robustness [45]. In terms of animal phenotypic measurement, early studies have confirmed that using short-range LiDAR/ToF depth sensors to obtain three-dimensional point clouds, combined with processes such as filtering, segmentation, and surface reconstruction, can achieve non-contact extraction of key body measurements of beef cattle, reaching high accuracy levels under controlled conditions [46]. However, common issues in actual farm environments, such as occlusion, hair surface scattering, posture changes, and point cloud noise, can significantly reduce the stability of key point localization and body measurement calculation. To address this, some studies have attempted to introduce prior knowledge or more stable acquisition and processing strategies to improve measurement reliability under low-quality point cloud conditions [47]. Building on the aforementioned research, Wang et al. [48] further addressed the problem of acquiring and automatically measuring three-dimensional data of beef cattle under natural feeding conditions by proposing a method for individual segmentation and automatic extraction of body measurements based on UAV-LiDAR point clouds. To tackle challenges such as freely moving beef cattle, frequent posture changes, and severe occlusion interference in open pasture environments, this study constructed a three-dimensional scanning system with multi-line LiDAR mounted on a UAV, enabling rapid acquisition and automatic processing of large-scale beef cattle point cloud data. As shown in Figure 5, the system obtains high-density three-dimensional point cloud data through the UAV platform and uses point cloud segmentation algorithms to identify individual beef cattle and calculate key body measurement parameters. Compared with fixed-channel RGB-D systems, LiDAR systems demonstrate higher stability and robustness in complex lighting and large-scale outdoor scenarios.

Building on the aforementioned two-dimensional image measurement, RGB-D measurement, and LiDAR measurement methods, this paper summarizes the application characteristics of these three major visual perception technologies in cattle body measurement to facilitate a more intuitive comparison. The analysis covers aspects such as spatial information acquisition capability, hardware cost, measurement accuracy, environmental adaptability, and data processing complexity. The results are presented in Table 2.

As shown in Table 2, there are significant differences among various visual perception technologies in terms of spatial information acquisition capability, hardware cost, measurement accuracy, and environmental adaptability. Two-dimensional imaging methods offer advantages such as low cost, simple deployment, and strong real-time performance; however, due to the lack of depth information, their ability to represent three-dimensional body measurements, such as chest circumference, body width, and body depth, is limited. RGB-D methods can simultaneously acquire color images and depth information, achieving a good balance between cost and accuracy; however, they are susceptible to depth noise under conditions of strong illumination, occlusion, and dynamic scenes. LiDAR-based methods can generate high-precision 3D point clouds and are insensitive to lighting variations, making them suitable for complex outdoor environments and large-scale pasture monitoring; however, they involve relatively high equipment costs, complex data processing, and challenging system integration. In practical pasture applications, factors such as lighting variation, animal movement, occlusion, and posture changes can lead to cumulative error propagation throughout the workflow of segmentation, keypoint localization, point cloud processing, and body measurement calculation. Therefore, the merits of a technology should not be evaluated solely on the basis of a single research result or measurement error; rather, an appropriate visual measurement solution should be selected by considering the measurement objectives, farming environment, cost constraints, and system maintenance requirements. It should be noted that Table 2 primarily compares single-vision perception approaches. As body measurement tasks place increasing demands on the completeness of 3D information and adaptability to complex environments, multi-view fusion systems are emerging as a promising direction for overcoming the limitations of single-view approaches.

### 3.3. Beef Cattle Body Measurement Method Based on Multi-View Fusion

To alleviate the common issues of occlusion and missing perspectives in single-view imaging or scanning for beef cattle body measurements, and to obtain more complete three-dimensional surface geometry information, multi-view fusion measurement systems have gradually become a research hotspot in recent years. Such systems usually deploy multiple depth sensors around the measurement space to synchronously acquire RGB-D images or point cloud data from different angles [49]. Through cross-view point cloud registration and fusion, a complete 3D model of the beef cattle is generated, thereby providing more sufficient geometric constraints for extracting body measurement parameters such as withers height, body length, and girth [50].

**Figure 6:**
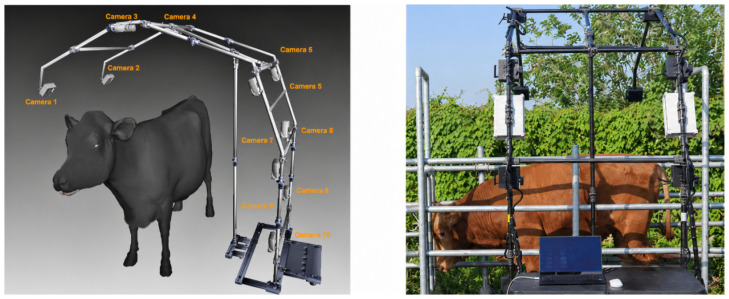
Deployment scheme of the multi-view RGB-D system. Reprinted from Reference [51], under the CC BY 4.0 license.

At the engineering deployment level, multi-view RGB-D systems typically consist of a synchronous triggering module, depth cameras, edge computing nodes, and a master computer to ensure comprehensive coverage of beef cattle’s backline, trunk contours, and lateral body surfaces. A typical configuration involves deploying multiple depth cameras along an aisle to enable synchronous data acquisition and point cloud fusion within a unified coordinate system. Dang et al. [51] developed a multi-view synchronous data acquisition system consisting of 10 Intel RealSense D435 depth cameras, which achieves temporal consistency of multi-view images through a hardware synchronization mechanism, as shown in Figure 6. The cameras are mounted on a fixed steel frame, enabling the simultaneous acquisition of depth information from multiple viewpoints of the cow’s body. Three-dimensional reconstruction of the cow’s body is achieved through multi-viewpoint point cloud fusion. This system effectively reduces occluded areas and improves point cloud completeness and reconstruction accuracy; however, it requires a large number of devices, entails high costs for system calibration and maintenance, and has certain requirements for the installation environment.

In terms of the technical workflow, multi-view fusion measurement systems typically follow the basic process of “multi-view synchronous data acquisition-sensor calibration-filtering and denoising-rough registration-fine registration-point cloud fusion-3D reconstruction-body measurement calculation.” Among these steps, the accuracy of cross-view point cloud registration and fusion directly affects the completeness of the 3D model and the stability of subsequent body measurement parameter calculations [36]. When there are temporal synchronization errors between cameras, extrinsic calibration errors, or insufficient overlap between point clouds, the fused surface of the beef cattle body may exhibit misalignment, ghosting, or local deformation, thereby affecting the measurement accuracy of indicators such as body height, body length, chest width, abdominal width, and girth. To facilitate comparison of differences among various multi-view systems in terms of sensor configuration, fusion strategies, and measurement accuracy, Table 3 summarizes representative multi-view body measurement studies from the past five years.

As shown in Table 3, existing multi-view measurement systems exhibit significant differences in sensor types, fusion methods, registration accuracy, and application scenarios. Compared to single-view measurement, the primary advantage of multi-view fusion lies in its ability to supplement information about the cow’s surface from different directions, thereby reducing errors caused by occlusion, pose changes, and missing local point clouds in single-view measurements. For example, Ruchay et al. [52] employed three RGB-D cameras to construct a non-rigid 3D shape measurement system. By fusing depth information from multiple viewpoints, they achieved high-precision measurements of body parameters, demonstrating that increasing the number of viewpoints in a controlled environment helps improve the completeness of 3D body surface reconstruction. Wu et al. [24] used three Zed2 stereo cameras and, under outdoor natural lighting conditions combined with a coarse-to-fine two-stage registration strategy, achieved average relative errors of 2.32% for body height and 2.27% for body length, indicating that multi-view point cloud fusion can mitigate the effects of complex lighting and single-view occlusion to a certain extent. Although the LiDAR-camera targetless calibration method proposed by Li et al. [53] achieved good results in terms of calibration accuracy, its adaptability in dynamic live-subject measurement scenarios remains to be verified. Du et al. [25] proposed a non-contact method for measuring livestock body dimensions based on multi-depth cameras. This method locates key anatomical landmarks on the animal’s body surface using a keypoint detection model and combines depth information to map 2D keypoints into 3D point cloud space, enabling the automated calculation of body dimension parameters and thereby improving the robustness and accuracy of measurements in complex environments.

However, performance improvements in multi-view fusion systems do not depend solely on an increase in the number of sensors; they are also influenced by a combination of factors, including camera placement, synchronized data acquisition, camera calibration, point cloud registration, and the stability of the fusion algorithms. Sensor placement must take into account field-of-view coverage, animal safety, and the spatial conditions of the pasture: if installed too low, sensors are prone to collisions with animals, contamination from manure, or obstruction; if installed too high, point cloud density in the flanks, abdomen, and leg areas may be reduced; narrow aisles limit the placement of side-view cameras, while wide aisles may reduce the ranging accuracy of depth cameras or result in insufficient point cloud resolution. Furthermore, dust, moisture, manure, and hair accumulation in the pasture environment can affect the imaging quality of lenses or depth sensors, requiring regular cleaning and maintenance; during long-term operation, equipment vibration, temperature fluctuations, gantry displacement, or loose cables may also cause drifts in sensor external parameters, gradually rendering the original calibration parameters invalid. Therefore, multi-view systems not only require precise calibration during initial installation but should also establish mechanisms for periodic calibration or online correction.

In summary, multi-view fusion measurement systems currently lack a unified, standardized approach. Significant differences exist across studies in terms of sensor configuration, fusion strategies, accuracy evaluation metrics, and testing environments, resulting in limited comparability of research findings. Future research should focus on two key areas: first, determining the appropriate number of viewing angles, sensor types, maintenance cycles, and deployment locations based on the measurement environment and target body dimensions to avoid reducing the system’s practical applicability due to excessive complexity; second, establishing a unified evaluation system for cross-view registration errors, body dimension measurement errors, and robustness under complex poses, thereby more objectively assessing the application potential of multi-view fusion systems in large-scale aquaculture scenarios.

## 4. Beef Cattle Body Measurement Extraction Algorithm Process and Technological Progress

### 4.1. Target Segmentation Methods in Beef Cattle Images

Target segmentation is the initial step in the visual measurement process. Its core task is to accurately separate the beef cattle target from complex farming backgrounds and provide a reliable foreground region for subsequent contour extraction, keypoint localization, point cloud registration, and body size calculation. The quality of segmentation directly determines the accuracy and stability of subsequent body size parameter extraction. If the segmentation result has boundary defects, local adhesion, or background residue, it will further amplify measurement errors of geometric parameters such as body length, body height, and chest depth [54]. Depending on the type of input data, existing studies generally divide target segmentation into two-dimensional image segmentation and three-dimensional point cloud segmentation. For two-dimensional images, research approaches mainly include traditional methods based on thresholding, edges, and background modeling, as well as deep learning methods based on semantic segmentation and instance segmentation [26]; for three-dimensional point clouds, target point cloud separation and structural representation are often achieved through geometric constraints, traditional feature learning, and deep learning networks such as the Point Transformer series [1]. Overall, image segmentation has gradually shifted from a traditional paradigm relying on manually designed features to a deep learning paradigm dominated by end-to-end learning. Semantic segmentation, instance segmentation, and attention-enhanced structures have become key technological foundations for contactless body size measurement of beef cattle.

#### 4.1.1. Two-Dimensional Image Segmentation

2D image segmentation is a key foundational step in the body measurement extraction process, with the goal of accurately separating the target area of beef cattle from the background in complex farming environments. The development of this technology has experienced a significant transition from traditional methods that rely on low-level visual features to end-to-end segmentation models based on deep convolutional neural networks, the latter showing stronger robustness and generalization capabilities in dealing with issues such as lighting variations, shadow interference, and dynamic backgrounds in complex farming environments. Traditional image segmentation methods usually depend on low-level visual features such as grayscale, color, texture, and edges. Typical methods include threshold segmentation, edge detection, and background modeling [55]. In video monitoring systems, threshold segmentation is widely used; for example, the Gaussian Mixture Model (GMM) can dynamically update the background and detect moving targets by establishing a probability distribution model of background pixels, thereby effectively separating foreground and background [56]. This method has low computational complexity and high real-time performance under fixed camera conditions, but it often relies on stable scene conditions and is sensitive to lighting changes, shadow interference, and complex dynamic backgrounds, thus limiting its robustness in actual farming environments [57]. With the rapid development of deep learning technology, convolutional neural networks have shown significant advantages in segmentation tasks in the field of agricultural vision, as they can automatically learn high-level semantic features of images and achieve pixel-level classification, making them more suitable for beef cattle segmentation tasks in complex farming environments. Among them, instance segmentation methods such as Mask R-CNN and semantic segmentation networks like DeepLabV3 have been widely applied in beef cattle image analysis tasks.

**Figure 7:**
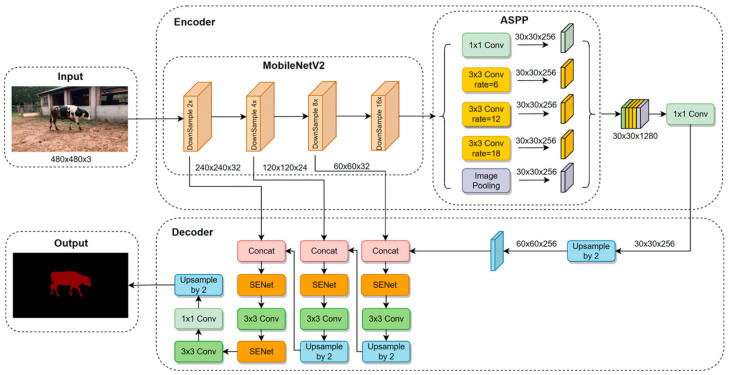
Schematic diagram of the improved DeepLabV3 beef cattle body segmentation network structure [60].

Bello et al. [58] proposed an Enhanced Mask R-CNN model to address the issues of blurred boundaries and irregular shapes of beef cattle in group farming environments. By introducing a multi-scale feature extraction mechanism and improving the region proposal network, this model effectively enhances the accuracy of multi-target beef cattle instance segmentation. In subsequent studies, Bello et al. [59] further optimized the model by integrating a transfer learning strategy, enabling the model to achieve better adaptability across multiple beef cattle datasets and significantly improving segmentation accuracy and computational efficiency. Similarly, Feng et al. [60] proposed a beef cattle body segmentation method based on an improved DeepLabV3 structure, as shown in Figure 7. This method uses MobileNetV2 as the backbone network and introduces an attention mechanism within the network to enhance feature representation. Experimental results indicate that the model can still achieve high-precision beef cattle semantic segmentation in complex multi-scenario environments, demonstrating good generalization capability. Xiao et al. [61] proposed an improved Mask R-CNN model for individual beef cattle identification in free-stall barn environments, effectively extracting back features of the beef cattle and enabling individual differentiation. Bello et al. [62] proposed an enhanced Mask R-CNN instance segmentation method. This method first applies the Generalized Color Fourier Descriptor (GCFD) for pre-enhancement of images, selects an optimal filter size smaller than ResNet101 to extract fine-grained and composite features, and uses the region proposal network to extract multi-scale semantic features. A sub-network is integrated into the original Mask R-CNN fully connected layer to improve segmentation accuracy, and the GrabCut algorithm is finally used for post-enhancement of images. Experiments on the cattle image dataset achieved a mean average precision (mAP) of 0.93, outperforming various existing methods and providing effective technical support for real-time individual beef cattle information acquisition in precision livestock farming. Qiao et al. [63] proposed a data augmentation technique based on random image cropping and patch expansion, expanding the number of training images and their corresponding labels, combined with advanced deep neural networks to achieve cattle image segmentation in farming environments. Experimental results show that this method achieves an average accuracy (mAcc) and mean intersection over union (mIoU) of 99.5% in complex backgrounds, significantly outperforming traditional augmentation methods such as random flipping, rotation, and color jitter, providing a feasible solution for automated segmentation in precision livestock farming. Zhang et al. [64] proposed a deep learning beef cattle detection method suitable for complex farm environments, showing stronger robustness under complex background conditions. In addition, with the development of foundation models, prompt-driven segmentation methods based on the Segment Anything Model (SAM) have also begun to be applied in agricultural vision tasks, demonstrating good potential in reducing annotation costs and improving model generalization [65].

In summary, 2D image segmentation technology has evolved from traditional methods reliant on low-level visual features to end-to-end models based on deep convolutional neural networks, significantly improving segmentation robustness and generalization capabilities in complex livestock farming environments. Methods such as Mask R-CNN and DeepLabV3+, through strategies including multi-scale feature fusion, attention mechanisms, and transfer learning, have effectively mitigated issues such as blurred boundaries and lighting interference in group-housing scenarios. With the advancement of foundational vision models, segmentation technology has further integrated with keypoint detection, multimodal perception, and unified visual representation frameworks, driving the intelligent evolution of precision livestock monitoring systems. However, it should be noted that the stability of segmentation results not only determines the quality of target region extraction but also exerts a persistent influence on subsequent keypoint localization and 3D reconstruction processes. This impact is progressively transmitted and amplified through the multi-stage body measurement calculation workflow, ultimately affecting the measurement accuracy of key body parameters such as height and chest circumference.

#### 4.1.2. 3D Point Cloud Segmentation

3D point cloud segmentation is a critical step in identifying and separating target objects based on spatial data acquired from depth cameras or LiDAR. Its core objective is to accurately extract point cloud regions corresponding to individual beef cattle in complex scenes. Compared to 2D images, 3D point clouds can more directly characterize the spatial structure and geometric shape of targets, giving them a distinct advantage in precision livestock applications such as body measurements, weight estimation, and posture analysis [66]. However, in actual livestock farming environments, point cloud segmentation still faces challenges such as ground noise, sparse point clouds, occlusion of individual animals, and overlapping multiple targets; these factors all affect the stability and accuracy of segmentation.

Based on their technical approaches, existing 3D point cloud segmentation methods can be broadly categorized into those based on geometric rules, those based on traditional feature learning, and those based on deep learning [67]. Early geometric rule-based methods typically relied on the spatial structure of the point cloud, using operations such as plane fitting, region growing, and DBSCAN to separate the foreground from the background. While these methods are relatively simple to implement and do not require large amounts of annotated data, they generally depend on scene assumptions and parameter settings, resulting in limited robustness in complex environments [68]. With the advancement of point cloud analysis methods, researchers have begun to utilize traditional features-such as normal vectors, curvature, and multiscale local geometric descriptions-for point cloud segmentation and classification. While these methods have improved the accuracy of point cloud object recognition to some extent, their cross-scene generalization capabilities remain limited due to their heavy reliance on feature engineering [69].

**Figure 8:**
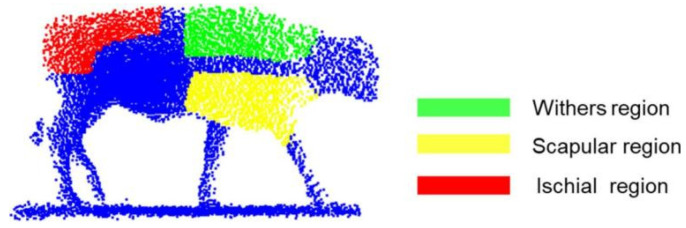
Deep Learning-Based Key Region Segmentation of Beef Cattle Point Cloud [75].

In recent years, deep learning has become the mainstream approach for 3D point cloud segmentation. Relevant studies have shown that methods based on direct point cloud learning, graph structure modeling, and attention mechanisms can more effectively extract local geometric features and global contextual information from point clouds, thereby improving segmentation performance in complex scenes [70,71,72,73,74]. In research on beef cattle body measurements, annotating and segmenting key regions in point cloud data allows for the relatively accurate localization of body measurement-related areas-such as body height and body length-providing a crucial foundation for subsequent calculations of body measurement parameters. Figure 8 shows the segmentation results of key regions in beef cattle point cloud data, where different colors correspond to different key body measurement locations on the cattle’s body surface. By automatically identifying and segmenting these regions, reliable spatial information can be provided for the extraction of parameters such as body height, body length, and backline [75].

With the continuous advancement of deep learning models, researchers have in recent years begun exploring the application of Transformer architectures, graph neural networks, and self-supervised learning methods to point cloud segmentation tasks, with the aim of further enhancing feature representation capabilities and model generalization in complex scenes. Table 4 summarizes and compares the characteristics of current representative 3D point cloud segmentation methods. In terms of model evolution trends, the research focus has gradually shifted from basic point cloud feature extraction to more robust contextual modeling and representation learning. Point Transformer enhances the ability to model point-to-point relationships through a self-attention mechanism [70], while Point Transformer V2 further refines vector attention and pooling strategies to improve large-scale point cloud segmentation performance [71]. Point NeXt improves the performance and scalability of classical point cloud networks through optimized training and scaling strategies [72]; furthermore, self-supervised pretraining methods such as Point-BERT and efficient graph neural network models like MLGCN provide new approaches for the generalization and deployment of point cloud segmentation in complex scenarios [72,73].

### 4.2. Method for Detecting Key Points on the Body Surface of Beef Cattle

Landmark detection is a core component of the beef cattle body measurement process. Its objective is to accurately locate anatomical landmarks or functional measurement points on the surface of the beef cattle or within a 3D point cloud for body measurement calculations, such as the highest point of the scapula, the chest circumference measurement point, the ischial tuberosity, and the highest point of the body. Compared to object segmentation, keypoint detection places greater emphasis on the precise localization of local structures; errors in this process directly affect the accuracy of subsequent body measurement calculations, and therefore plays a crucial role in automated measurement systems [76].

**Figure 9:**
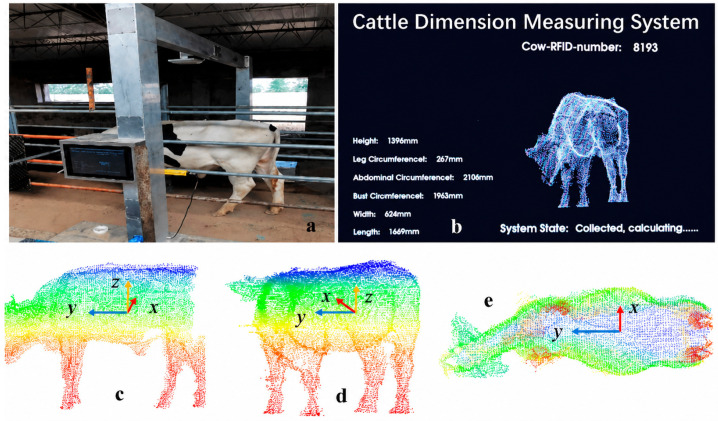
Schematic diagram of the beef cattle point cloud data acquisition system [78]: (**a**) collection device; (**b**) point cloud data collection system; (**c**) front view; (**d**) left view; (**e**) top view. The color gradient represents spatial depth information, and different colors correspond to height variations in the 3D coordinate system.

In recent years, researchers have proposed various methods for keypoint localization, including those based on geometric features and deep learning. These methods typically rely on high-quality 3D point cloud data, which is primarily acquired using 3D vision capture devices [77]. The beef cattle point cloud acquisition device described in detail by Li et al. [78] collected 3D point clouds from 10 male Holstein beef cattle aged 11–13 months. All data were automatically collected as the cattle passed through the device. A total of 182 sets of point clouds were collected from beef cattle in various poses. Detailed information about the experimental acquisition device and examples of the collected point cloud data are shown in Figure 9.

#### 4.2.1. Geometric Feature Method

Keypoint detection methods based on geometric features represent one of the earliest technical approaches developed in the field of 3D measurement of beef cattle. Building upon point cloud segmentation, denoising, and pose normalization, these methods utilize geometric features-such as contour variations, curvature distributions, and local extrema-in the 3D space of the beef cattle’s body to establish a mapping relationship between anatomical structures and measurement points [79]. Compared to data-driven methods, these approaches do not rely on large-scale annotated samples, offering high interpretability and low computational complexity. However, their performance is highly dependent on point cloud quality and pose stability, and they are prone to errors under conditions of occlusion or pose changes [80].

**Figure 10:**
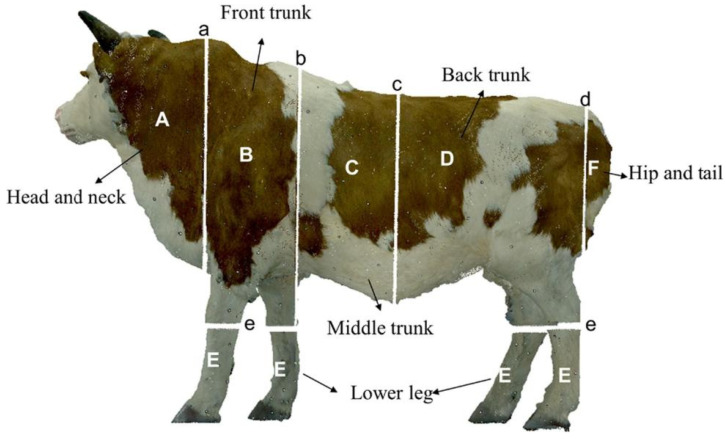
Beef Cattle Segmentation Plan [81].The cattle body is partitioned into anatomically meaningful regions for geometric measurement. A–F represent head and neck (A), front trunk (B), middle trunk (C), back trunk (D), hip and tail (F), and lower leg (E), respectively. This anatomical segmentation enables robust region-based feature extraction and improves posture-adaptive measurement consistency.

Among geometric feature-based methods, key region extraction based on tomographic slices has emerged as an important research direction in recent years. Li et al. [81] proposed the Bidirectional Tomographic Slice Segmentation (BTSS) method, which constructs forward and backward slice sequences along the main axis of the beef cattle body. By analyzing the geometric changes between consecutive slices, this method automatically segments key regions such as the head and neck, forequarters, mid-body, and hindquarters, as shown in Figure 10. This method effectively addresses the stability issues associated with traditional region segmentation strategies based on single extrema or local contour changes, and achieved a region recognition accuracy of approximately 92.8% in experiments, providing a stable structural foundation for subsequent keypoint localization.

However, relying solely on static geometric structures for localization remains inadequate for accommodating the effects of changes in animal posture. To address this issue, Li et al. [27] further proposed a posture-based measurement correction method, which adjusts body measurement results by incorporating micro-posture parameters of the beef cattle’s head, torso, and limbs, thereby improving measurement consistency under different posture conditions. This study indicates that the primary sources of error in geometric methods stem not only from point cloud noise but are also closely related to structural offsets caused by posture changes; therefore, introducing posture constraints based on key region extraction is an effective means of improving measurement stability.

In addition to slice-driven regional analysis methods, early studies also widely employed extreme value or projection geometry methods to estimate key anatomical locations. These methods typically approximate the highest or widest points of the body by analyzing the maximum or minimum values of the point cloud in specific directions. While they offer advantages such as simplicity and computational efficiency, they are relatively sensitive to pose variations. To mitigate this issue, Du et al. [25] proposed a body measurement method based on multiple depth cameras, which first detects the positions of key points in two-dimensional images and then combines depth information to reconstruct their three-dimensional coordinates, thereby enabling the automatic calculation of multiple body measurement parameters. While this method reduces reliance on complete 3D geometric structures to some extent, its measurement accuracy remains influenced by both 2D detection errors and the quality of depth information.

As research has progressed, keypoint detection has gradually evolved from rule-based geometric methods to data-driven approaches. Li et al. [82] proposed combining 2D keypoint detection with monocular depth estimation to achieve automatic measurement of beef cattle body dimensions, demonstrating good adaptability under complex acquisition conditions. Furthermore, Yang et al. [83] constructed a single-stage end-to-end keypoint detection network, unifying keypoint localization and body measurement calculation within a single framework, thereby significantly enhancing the automation of the measurement process. Therefore, from the perspective of methodological trends, geometric feature-based methods are gradually converging with deep learning methods, transforming from independent rule-based systems into integral components that provide structural constraints.

#### 4.2.2. Methods Based on Deep Learning

Deep learning-based keypoint detection methods train neural networks to automatically learn the spatial distribution patterns of anatomical landmarks on beef cattle and directly output the position coordinates of keypoints or their probability heatmaps. Compared to methods based on geometric rules, these approaches can learn complex morphological features from large datasets and therefore typically exhibit greater robustness under conditions of complex backgrounds, pose variations, and partial occlusion. In recent years, with the rapid development of deep learning in the field of pose estimation, keypoint detection technology has become one of the major research directions in automatic body measurement. Relevant studies have shown that deep learning-based keypoint detection methods outperform traditional geometric methods in terms of accuracy, stability, and automation [82].

Based on differences in input data types and system architecture, existing deep learning-based keypoint detection methods can be classified into three categories: methods based on 2D images, methods based on 2D–3D fusion, and methods based on 3D point clouds. Table 5 summarizes the main method frameworks and their characteristics from relevant research over the past five years.

For keypoint detection methods in 2D images, researchers typically draw on the concepts of heatmap regression or coordinate regression from human pose estimation to predict the locations of keypoints in images using convolutional neural networks. For example, the high-resolution network HRNet achieves accurate keypoint localization by preserving multiscale, high-resolution feature representations, and has demonstrated strong performance in various pose estimation tasks. In their benchmark study on precision livestock farming, Wang et al. [84] compared various keypoint detection frameworks, including SimCC, SAR, and YOLOX-Pose, and noted that SimCC-HRNet-W48 performed best in terms of keypoint localization accuracy, while YOLOX-Pose had advantages in terms of speed and model compactness. Furthermore, some studies have performed model lightweighting or structural optimization of keypoint detection networks to address complex backgrounds and occlusion issues in livestock farm environments. For example, Li et al. [85] proposed an improved beef cattle pose estimation network based on RTMPose, enhancing the real-time performance of keypoint detection by introducing a more efficient feature extraction module. Zhao et al. [86] proposed a lightweight cattle pose estimation network that improves keypoint detection accuracy in complex scenes through a reparameterized architecture and attention mechanisms.

However, obtaining keypoint coordinates from two-dimensional images alone cannot be directly applied to body measurements, as metrics such as height and length are inherently three-dimensional geometric quantities. Consequently, in recent years, a large body of research has begun to explore methods for fusing 2D keypoint detection with 3D point cloud data. Within this framework, keypoints are first detected in RGB images and then mapped to 3D space using depth information or camera calibration parameters, thereby obtaining the 3D coordinates of keypoints on beef cattle. Zhao et al. [87], using RGB-D data acquired from a RealSense D455 depth camera, employed the Deep Lab Cut deep learning framework for keypoint detection and calculated geometric parameters via a dynamic depth substitution optimization method. This approach enabled high-precision, real-time acquisition of beef cattle body measurement parameters, thereby improving grouping and management efficiency in large-scale farming operations.

In addition to 2D–3D fusion methods, another class of research attempts to perform keypoint detection directly within 3D point clouds. These methods typically rely on deep learning models for point clouds, which predict keypoint locations by learning the spatial structural features of the point cloud. For example, some studies utilize keypoint detection networks combined with point cloud reconstruction or depth completion techniques to achieve non-contact body measurement. Yue et al. [28] proposed an automatic measurement method combining YOLOv8-Pose with 3D point clouds; by detecting key points in 2D images and combining them with point cloud data for 3D localization, they achieved low body measurement errors. Weng et al. [88] further proposed the YOLOv8-DMC method, which combines key point detection with 3D point cloud reconstruction to achieve non-contact automatic measurement of beef cattle body dimensions. Additionally, Xu et al. [89] proposed a 3D keypoint detection method based on geodesic distance regression, which achieves point cloud keypoint localization through an improved PointNet++ network and yields good results in animal body measurement tasks.

Overall, deep learning-based keypoint detection methods are gradually evolving from standalone 2D pose estimation toward 2D–3D fusion and end-to-end measurement systems. In current research, 2D keypoint detection methods have reached a relatively mature stage, while 2D–3D fusion methods demonstrate high engineering feasibility in automated body measurement systems; in contrast, keypoint detection directly based on 3D point clouds remains in the developmental stage but holds significant research potential.

### 4.3. 3D Reconstruction and Pose Alignment

In livestock phenotyping research, although two-dimensional imaging methods have made some progress, the lack of depth information makes the results susceptible to factors such as changes in viewing angle, perspective distortion, animal posture, and occlusion. This makes it difficult to accurately reconstruct the animal’s true spatial structure, thereby limiting measurement accuracy and stability [90]. In recent years, with the development of 3D vision technology, 3D reconstruction methods have gradually become an important technical tool for automated livestock phenotyping. By reconstructing the three-dimensional geometric structure of an animal’s body surface, key morphological parameters such as body length, height, chest circumference, and volume can be obtained more accurately, thereby significantly improving the reliability and precision of automated body measurement systems [76].

In practical applications, 3D reconstruction technology typically involves three key steps: 3D model reconstruction, point cloud preprocessing, and pose normalization. First, the raw point cloud data must undergo filtering and denoising to improve data quality; second, the three-dimensional structure of the animal’s body surface is captured using multi-view geometric or depth sensors; and finally, the effects of changes in the animal’s posture are eliminated through pose alignment methods, thereby ensuring measurement consistency across different samples [91].

#### 4.3.1. 3D Reconstruction

**Figure 11:**
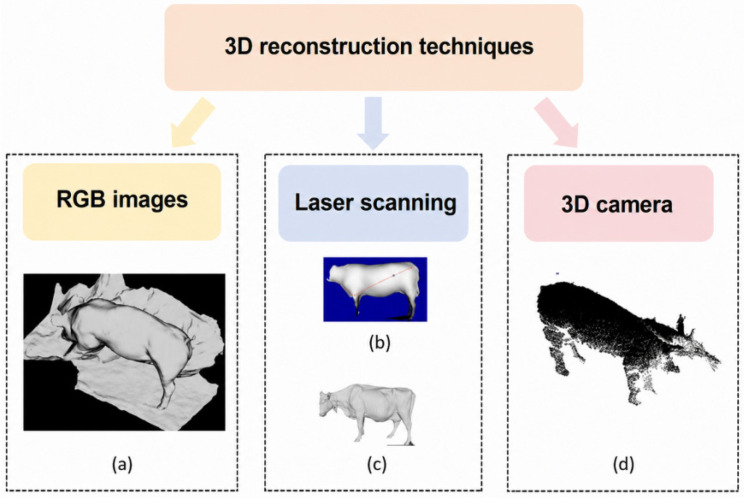
Three different 3D reconstruction methods based on RGB images, laser scanning, and 3D cameras Reprinted from Reference [44], under the CC BY 4.0 license. (**a**) Demonstrates 3D reconstruction technology using RGB images; (**b**,**c**) reconstructed using a laser scanning; (**d**) reconstructed using a 3D camera.

Currently, 3D reconstruction methods in livestock visual measurement can be broadly categorized into three types: 3D reconstruction based on multi-view images, reconstruction based on laser scanning, and reconstruction based on 3D cameras. Different visual technologies exhibit significant differences in measurement accuracy, cost, and deployment complexity; a comparison of their overall technical approaches and performance is shown in Figure 11 [44]. These technologies provide innovative computer vision methods for accurate and non-contact measurement of livestock body conformation and weigh.

Multi-view 3D reconstruction methods capture images of animals from multiple cameras at different viewpoints and use multi-view geometric algorithms to reconstruct the target’s 3D structure. Typical methods include Structure-from-Motion (SfM) and Multi-View Stereo (MVS) [51]. These methods generate dense 3D point cloud models by matching feature points in the images and using triangulation to calculate spatial coordinates. In recent years, some studies have utilized multiple depth cameras to construct multi-view fusion systems, enabling complete 3D reconstruction of beef cattle body surfaces. The multi-view depth fusion method proposed by Li et al. [35] can reconstruct a complete body surface model of beef cattle and perform automated body measurements, providing important technical support for intelligent livestock monitoring systems.

Laser scanning technology, with its non-contact and high-precision characteristics, has become one of the core technical approaches for live body measurements of beef cattle. Huang et al. [92] used LiDAR sensors to collect live point cloud data from Qinchuan beef cattle. By employing a multi-stage filtering algorithm to eliminate background noise and combining ICP point cloud registration with surface reconstruction techniques, they achieved automated measurement of key body dimensions such as height at withers and body length. This approach addressed the issues associated with traditional manual measurement-including high stress levels, low efficiency, and significant subjective errors-and provided an objective and efficient technical means for beef cattle growth monitoring and breeding evaluation; Lu et al. [93] further proposed a method for measuring beef cattle body dimensions based on the fusion of graph convolutional networks and parametric models. Through a two-stage coarse-to-fine alignment strategy, they achieved the accurate extraction of nine indicators-including shoulder height and chest circumference-in complex grazing scenarios, with the average absolute percentage error controlled within 3.58%, reducing the measurement process for a single beef cow to within 30 s and providing a more efficient and robust solution for real-time phenotypic analysis on large-scale ranches.

With the advancement of depth sensor technology, RGB-D cameras have gradually become essential devices in automated livestock monitoring systems. Common devices include the Microsoft Kinect and Intel RealSense. Ruchay et al. [52] used three Microsoft Kinect v2 depth cameras, combined with a non-rigid 3D shape reconstruction algorithm, to achieve automated, non-contact measurement of nine body measurements in beef cattle, thereby addressing the issues of time-consuming manual measurement and high stress levels. Meanwhile, Kamchen et al. [94] utilized an Intel RealSense D435i depth sensor, combined with image analysis and linear regression models, to estimate the body weight and multiple body measurements of young Nellore beef heifers, thereby validating the application potential of RGB-D sensors in beef cattle phenotyping. Such devices can directly capture depth information of the target, enabling rapid generation of 3D point cloud data. Compared to traditional stereo vision systems, depth camera systems have a simpler structure and can perform real-time 3D data acquisition, making them highly valuable for application in automated livestock farming environments.

In summary, the three categories of 3D reconstruction methods in livestock vision measurement-multi-view RGB reconstruction, laser scanning, and RGB-D depth sensing-essentially represent a trade-off between accuracy, complexity, and scene adaptability. The multi-view method relies on passive vision and feature matching; while it can achieve robust reconstruction under static, texture-rich conditions, it is sensitive to occlusion and dynamic deformation, is computationally intensive, and is difficult to implement in real time. Laser scanning uses active ranging to acquire high-density, high-precision point clouds and offers benchmark-level advantages in structured light and morphometric applications; however, its high cost and demanding data acquisition conditions limit large-scale deployment in grazing environments. RGB-D cameras effectively reduce the front-end complexity of 3D reconstruction by directly acquiring depth information and supporting real-time data streams; however, depth quality is easily affected by strong light, distance, and target surface properties, resulting in an upper limit on accuracy. These three technologies do not follow a linear iterative relationship but rather represent complementary approaches tailored to different measurement tasks and constraints. Future efforts should aim to overcome the limitations of single-sensor systems by developing online 3D perception methods that integrate multiple data sources, are lightweight, and can adapt to dynamic deformations, thereby enabling continuous, high-fidelity digital representation of livestock body shapes.

#### 4.3.2. 3D Preprocessing and Pose Alignment

**Figure 12:**
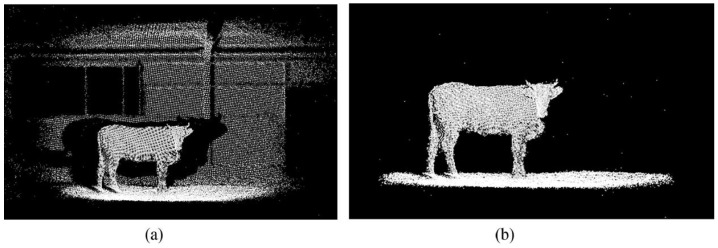
Comparison of Point Cloud Before and After Denoising [28]: (**a**) is the original point; (**b**) is the point after filtering.

Raw point cloud data is generally affected by factors such as sensor measurement errors, changes in ambient lighting, and spontaneous animal movement, resulting in issues such as noise points, outliers, and missing data in localized areas [95]. The core objective of point cloud preprocessing is to effectively remove abnormal noise points while preserving the main geometric structure of the target animal and retaining its primary geometric features, as shown in Figure 12 [28], thereby providing a high-quality data foundation for subsequent 3D modeling. Weales et al. [95] proposed a robust machine vision system for beef calves, which achieved body measurement in complex farming environments through multi-view data acquisition and point cloud segmentation techniques. Wang et al.’s [76] systematic review analyzed 47 applications of 3D vision in beef cattle growth management, noting that point cloud clustering and conditional filtering are currently the most mainstream noise removal methods, while also pointing out that existing methods still face challenges when handling dynamic scenes and occlusion issues. Regarding the evolution of denoising methods, Zhao et al. [96] proposed a point cloud denoising algorithm combining improved radius filtering with local plane fitting. Through an adaptive neighborhood selection strategy, this method effectively balances the trade-off between noise removal and feature preservation, achieving an average accuracy of 96.37% under a 40% noise ratio-significantly outperforming traditional KNN methods. Notably, Retta et al. [97] developed the CattleDeSegNet joint model, which integrates point cloud denoising with the interpretable segmentation task within a single network framework, enabling end-to-end processing for cattle point clouds. In terms of data compression and computational efficiency optimization, Lyu et al. [98] proposed a dynamic point cloud downsampling algorithm based on voxel filtering. This method can adaptively adjust the sampling density according to local features of the point cloud, improving processing speed by approximately two orders of magnitude compared to traditional methods while effectively preserving geometric details in edge regions. To address the issue of point cloud data integrity restoration, Gai et al. [99] proposed a point cloud hole-filling algorithm based on a motion recovery structure. This method first extracts hole boundaries using two-dimensional phase stripe projection and then fits and fills the missing regions using radial basis functions, thereby achieving comprehensive restoration of holes in complex surfaces.

After obtaining a complete 3D reconstruction model, pose normalization is an indispensable step prior to body measurement calculation. Due to individual variations in body tilt, head orientation, and standing posture during data acquisition, the 3D models of different samples exhibit different spatial orientations. If body measurements are calculated directly based on the original poses, these pose differences will be introduced into the measurement results as systematic errors, severely affecting the accuracy and comparability of the body measurement parameters. Wang et al. [48] proposed an automated method for extracting beef cattle body measurements based on UAV-mounted LiDAR and innovatively introduced a dual-rotation algorithm to perform pose normalization for cattle head orientation. By using multivariate analysis of variance to remove outlier data points, they effectively improved the reliability of the measurement results. Luo et al. [100] proposed an automatic livestock body measurement method based on statistical shape models. By fitting a parametric statistical shape model to the point cloud data, they addressed the issue of data loss caused by self-occlusion in the point cloud while simultaneously performing pose normalization, achieving an overall accuracy of 91.95% in beef cattle measurements.

Currently, mainstream pose normalization methods are primarily divided into two categories: those based on principal component analysis (PCA) and those based on keypoints. PCA-based pose alignment is one of the commonly used approaches. This method calculates the three-dimensional spatial covariance matrix of the point cloud, extracts the principal direction vector as the object’s principal axis, and aligns the animal’s body axis with the standard coordinate axes. It offers advantages such as simplicity of implementation and high computational efficiency [74]. However, this method also has significant limitations. When the animal’s body is severely twisted or deformed, or when the point cloud contains a large number of missing regions, the calculation of the principal direction is highly susceptible to outliers, leading to significant deviations in the alignment results. Furthermore, the PCA method relies solely on the statistical characteristics of global geometric information and cannot effectively utilize anatomical information from the animal’s body surface; consequently, it faces accuracy limitations in scenarios involving significant postural deformation. Li et al. [27] proposed a posture-based measurement adjustment method. By analyzing the patterns of how a cow’s standing posture affects body measurements, they established a mapping relationship between posture parameters and measurement errors. After normalizing the posture, they corrected and compensated for the measurement results, effectively reducing the systematic bias introduced by posture variations. To overcome the limitations of the PCA method, researchers have turned to keypoint-based pose correction methods. The theoretical core of this approach involves detecting several key anatomical points on the animal’s body surface and using these anatomically significant landmarks to construct a local reference coordinate system, thereby achieving higher-precision pose normalization. Yang et al. [101] combined keypoint detection with local point cloud clustering to construct an end-to-end automated body measurement workflow for cattle. By introducing the YOLOv8-SimBiFPN keypoint detection model, they controlled the average absolute percentage error of shoulder height to 4.37% in the free-standing posture. Zhou et al. [102] achieved pixel-level semantic segmentation of dairy cow point clouds using the FGPointKAN++ model and, in combination with adaptive key-plane identification technology, performed automatic measurement of body conformation parameters under various walking postures. Guo et al. [103] proposed a posture normalization framework based on bilateral symmetry, which utilizes the natural left-right symmetry of the livestock’s body surface to construct a reference coordinate system. This method does not rely on keypoint detection but instead determines posture by identifying the symmetry plane of the point cloud. Experiments confirmed that this framework significantly outperforms the traditional PCA benchmark algorithm in terms of posture normalization performance.

In summary, 3D reconstruction technology provides fundamental spatial geometric information for animal body measurement; however, factors such as spontaneous animal movement, localized point cloud gaps, and complex background interference in actual farming environments continue to limit further improvements in measurement accuracy. The integrated application of point cloud preprocessing, restoration, and pose normalization techniques-combined with the selection of appropriate method combinations based on specific application scenarios-is the key approach to improving the accuracy of vision-based animal body measurement.

### 4.4. Body Measurement Parameter Calculation

After completing target segmentation, keypoint detection, and 3D reconstruction, the calculation of body measurements converts visual data into quantifiable animal phenotypic indicators, serving as a critical link between perceptual information and precise evaluation. Compared to front-end visual tasks, this process relies more heavily on the accuracy of scale restoration and the soundness of geometric modeling; errors typically stem from segmentation deviations, keypoint localization errors, and the cumulative effects of depth information noise [92]. From an overall workflow perspective, existing studies generally follow a unified framework of “structure extraction–scale mapping–geometric computation.” First, the body contour or key structures of beef cattle are extracted through segmentation or keypoint detection; second, camera calibration parameters or depth information are used to convert pixel-scale measurements to real-world scale; and finally, metrics such as body height, body length, and girth are calculated based on Euclidean distance, cross-sectional perimeter, or spatial geometric relationships [29].

Early methods were primarily based on geometric models, using two-dimensional contours or three-dimensional surfaces to construct explicit geometric relationships for body measurement calculations. Lu et al. [93] proposed an automatic beef cattle body measurement method based on the fusion of an improved graph convolutional network and a 3D parametric model, achieving accurate extraction of nine body measurement indicators through a two-stage coarse-to-fine alignment strategy. Zhang et al. [104] proposed a method for single-sided beef cattle point cloud restoration and body measurement extraction based on moving least squares. They preprocessed the point cloud using Euclidean clustering, conditional filtering, and the random sampling consistency algorithm; used least-squares deformation to repair holes caused by camera viewing angle limitations; and employed polynomial fitting and cubic B-spline curve fitting to automatically calculate five parameters, including body height, chest circumference, and body length. Geometric modeling methods offer advantages such as simple structure, high computational efficiency, and strong interpretability. However, due to the non-rigid nature of an animal’s body surface and the presence of pose changes and occlusion issues in real-world acquisition environments, their measurement accuracy is often limited in complex scenarios.

With the advancement of 3D vision technology, research has gradually shifted toward computational methods that make fuller use of spatial information. Reconstructing the 3D structure of beef cattle using depth information or point cloud data, and estimating body measurements based on spatial geometric features, can effectively improve measurement accuracy and stability. Wang et al. [48] proposed an automated method for extracting beef cattle body measurements based on UAV-mounted LiDAR point clouds. Point cloud data were acquired through scanning under natural rearing conditions, and body measurement parameters were extracted by identifying surface landmarks through an automated segmentation process. To further improve accuracy, the study incorporated multivariate analysis of variance to adjust the dataset, excluding data collected at flight altitudes of 30 m and 50 m and flight speeds of 7 m/s and 9 m/s, which significantly reduced measurement errors. Concurrently, a linear regression model with 10-fold cross-validation was established using 136 training data pairs of withers height and hip height, enabling precise estimation of anatomical hip height. Additionally, a dual-rotation algorithm was proposed to normalize the head orientation of cattle. However, purely 3D methods are highly dependent on point cloud quality; when point cloud density is insufficient or noise levels are high, both surface reconstruction and geometric measurement results are compromised.

Building on this, a large number of studies in recent years have further introduced keypoint detection methods. By locating anatomical landmarks such as the acromion, ischial tuberosity, and the highest point on the back, these methods directly calculate body measurement parameters using the spatial relationships between keypoints. This approach significantly enhances automation and reduces reliance on a complete contour. Weng et al. [105] proposed a beef cattle body measurement system based on DUOS-PointNet++. Using a dynamic unbalanced octree clustering algorithm, they performed pixel-level semantic segmentation on the cattle point cloud, dividing the cattle body into seven sections: body length, shoulder height, rump height, chest circumference, abdominal circumference, and cannon bone circumference. Combining techniques such as density measurement, point cloud slicing, contour extraction, and point cloud restoration, the system achieved measurement accuracies of 1.18% relative error for shoulder height, 1.34% for rump height, 2.52% for body length, and 2.12% for chest circumference on a dataset of 137 cattle. Weng et al. [88] proposed the YOLOv8-DMC lightweight deep learning model, specifically optimized for the detection of anatomical landmarks in side-view images of beef cattle. This model integrates three attention modules—DRAMiTransformer, MHSA-C2f, and CASimAM—to enhance robustness under occlusion and varying lighting conditions. After keypoint prediction, a color point cloud is generated through 16-neighborhood depth interpolation and smoothing filtering to achieve precise 3D localization of the keypoints.

**Figure 13:**
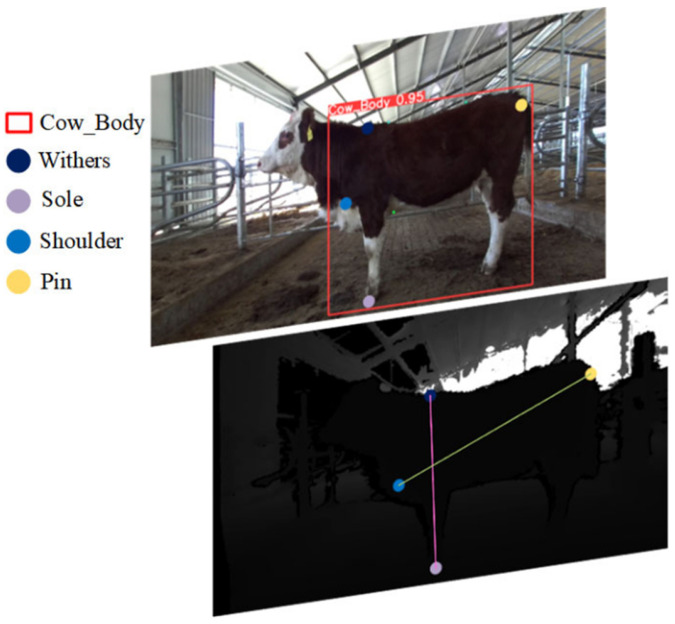
Schematic Diagram of Body Length and Height Calculation. Reprinted from Reference [40], under the CC BY 4.0 license. In the diagram, the green dot denotes the anatomical reference keypoint used for body measurement calculation.

Furthermore, to improve measurement accuracy and robustness, research typically combines key point detection with depth information to map key points from two-dimensional images to three-dimensional space, thereby obtaining more accurate body measurement estimates. A typical key-point-driven body measurement calculation process is shown in Figure 13, where the spatial relationships among key points are directly used to construct measurement metrics such as body length and height.

In order to systematically compare the characteristics and applicability of different body measurement calculation methods, Table 5 summarizes mainstream methods in terms of data format, calculation approach, and performance.

As shown in Table 6, there are significant differences among various body size calculation methods in terms of accuracy, computational complexity, and applicable scenarios. Current research trends are gradually shifting toward the deep integration of keypoints and 3D information, aiming to achieve more stable and accurate body size measurements in complex aquaculture environments by combining structural semantic information with spatial geometric information. Furthermore, errors in body size estimation primarily stem from factors such as inaccurate segmentation, keypoint localization deviations, depth information noise, and changes in animal posture. Therefore, incorporating posture normalization, multi-frame fusion, and error correction mechanisms during the estimation phase is a key approach to improving the system’s overall measurement performance.

## 5. Conclusions—Application Challenges in Complex Pasture Environments

### 5.1. Lighting Variations, Animal Movement, and Occlusion Interference

In complex pasture environments, lighting variations, animal movement, occlusion interference, and posture changes are the primary factors affecting the stability of visual body measurement systems. These factors do not merely degrade image quality; rather, they introduce errors that propagate step by step through the “image acquisition-object segmentation-keypoint localization-3D reconstruction/point cloud registration-body measurement calculation,” ultimately affecting the measurement accuracy of body parameters such as height, length, chest width, chest depth, chest circumference, and abdominal circumference. Therefore, complex environmental factors should be regarded as sources of error throughout the entire visual body measurement process [106,107,108,109].

Changes in lighting are the most common source of interference in field applications. Direct sunlight, shadows, backlighting, localized overexposure, insufficient nighttime lighting, and variations in brightness inside and outside the barn can alter the color, texture, and edge contrast of the cow’s body surface, diminishing the visual distinction between the cow and the background. This leads to issues in object segmentation, such as boundary gaps, residual background, or localized adhesion, which in turn affect the extraction of parameters based on contours or geometric boundaries-such as body length, height, and chest depth [78]. In keypoint detection, strong shadows or local overexposure may also obscure anatomical features such as the acromion, hip, topline, and thoracoabdominal contours, causing shifts in keypoint localization. For RGB-D and multi-view 3D measurement systems, strong light may interfere with the active signals of structured light or ToF depth sensors, causing gaps, noise, and local distortions in the depth map; exposure differences between different viewpoints can also reduce the accuracy of point cloud registration and fusion, ultimately affecting the integrity of the 3D model and the calculation of parameters such as chest circumference, abdominal circumference, body width, and body depth [88].

Animal movement, occlusion, and postural changes can also affect measurement results. When beef cattle walk naturally, turn their heads, wag their tails, lower their heads to feed, or adjust their posture, this can cause motion blur in images and temporal inconsistencies in point clouds across multiple frames, leading to instability in segmentation boundaries, keypoint locations, and cross-view registration results [110]. Occlusion caused by railings, feed troughs, fences, other beef cattle, and the cattle’s own body parts can lead to the merging of target regions, the invisibility of keypoints, and local point cloud gaps, which particularly affect the estimation of 3D parameters in areas such as the chest, abdomen, hindquarters, and lower abdomen. Furthermore, non-standard postures-such as lowering the head, turning the head, arching the back, standing at an angle, and taking steps-can alter the body contour and the spatial positions of keypoints, resulting in deviations between automatic measurements and manual standard measurements [46]. To address these issues, future research could focus on collaborative improvements across data acquisition, algorithms, and measurement, including fixed measurement channels, light shielding and uniform illumination, synchronized triggering, multi-view setup, high-frame-rate acquisition, lighting normalization, shadow suppression, motion blur enhancement, occlusion enhancement, pose estimation, temporal filtering, and multimodal fusion, to improve the robustness and reliability of visual body measurement systems in real-world pasture environments [106].

### 5.2. Needs for Public and Standardized Annotations

Public datasets form the foundation for the reproducible evaluation and cross-comparison of visual body measurement algorithms. It should be noted that while there is not a complete lack of open data related to beef cattle vision in the current field, there is a shortage of public benchmark datasets specifically designed for beef cattle body measurement tasks that simultaneously incorporate multimodal data, standardized body measurement labels, individual metadata, and data collected across different pastures [111]. In recent years, the field of precision livestock farming has seen the emergence of several open data resources related to bovine visual phenotyping, body condition scoring, live weight estimation, pose estimation, and individual identification [112,113]. Winkler, Boucheron et al. [114] released the Criollo beef cattle RGB-D body condition scoring dataset, which includes pre-classified RGB-D videos as well as processed depth maps, grayscale images, and edge maps, and can be used for beef cattle body condition scoring and 3D visual phenotyping studies. The CowDatabase, constructed by Ruchay et al. [51], contains multi-view RGB-D data, point clouds, and manual body measurements for 103 Hereford cattle, which can be used for 3D reconstruction of live beef cattle and the measurement of morphological parameters. Subsequent studies also utilized a dataset comprising RGB-D images, live weights, and manual measurements for 154 Hereford beef cattle to predict live weight [41]. The BECA dataset proposed by Zhang et al. [113] consists of BECA-D, which focuses on visual diversity across multiple breeds, and BECA-L, which is designed for long-term individual identification. MultiCamCows2024, released by Yu et al. [112], contains multi-camera images and surveillance videos of 90 Holstein-Friesian cows on a working dairy farm and can be used for individual cow identification and multi-camera livestock monitoring.

These datasets provide an important foundation for research on visual perception in beef cattle, but there are still gaps between them and the “Automatic Body Measurement Reference Dataset”. First, many datasets are primarily designed for body condition scoring, live weight estimation, pose estimation, or individual identification, and do not systematically provide standard body measurement labels such as height, body length, chest width, chest depth, abdominal width, hip width, chest circumference, and abdominal circumference. Second, some datasets contain only RGB images, depth maps, or individual identification labels, lacking RGB-D data, LiDAR point clouds, multi-view images, camera calibration parameters, and synchronized acquisition information, making it difficult to support 3D reconstruction, cross-modal comparisons, and complete body measurement workflows. Third, existing data often focus on specific farms, breeds, sensors, or tasks, making it difficult to evaluate model stability under varying pen structures, lighting conditions, background interference, breed body types, and equipment configurations. Fourth, inconsistencies in data access methods, licensing agreements, data segmentation, and evaluation metrics make it difficult to conduct fair and reproducible comparisons between different research results. A review of existing precision livestock vision datasets also points out that, although the number of publicly available livestock vision datasets is increasing-and a significant portion of them are beef cattle-related-the lack of high-quality, multi-environment, multi-animal, and multi-task annotated datasets, as well as the absence of contextual metadata, remains a major bottleneck limiting the field’s development.

Therefore, future public datasets for beef cattle body-scale visual measurement should shift from “providing images” to “providing reproducible experimental benchmarks”. Specifically, datasets should include at least five categories of core information. First is sample size and collection structure, including the number of farms, the number of beef cattle, the number of image or video frames, the number of point clouds, the collection cycle, the number of repeated measurements, and the sample distribution per cow. Simply reporting the number of images is insufficient, as adjacent frames in continuous video are highly similar; without specifying the number of beef cattle and the acquisition time, it is easy to overestimate the effective diversity of the dataset. Second is animal metadata, including breed, age, sex, growth stage, weight range, body condition score, health status, and feeding method. Beef cattle body conformation, coat color and texture, skeletal development, and changes in body condition can all affect the generalization ability of visual measurement models. Third is sensor and acquisition conditions, including RGB images, depth maps, RGB-D data, stereo images, LiDAR point clouds, multi-view videos, weighing data, sensor models, camera mounting height, shooting angles, indoor and outdoor environments, lighting conditions, and whether acquisition is conducted in a walk-through or open-access setting. For 3D measurement tasks, camera intrinsic and extrinsic parameters, depth calibration, scale references, point cloud registration parameters, and time synchronization information should also be provided. Fourth is annotation content and quality control; in addition to bounding boxes and segmentation masks, anatomical landmarks, individual IDs, pose labels, body weight, body condition scores, and manually measured body dimensions should be provided whenever possible. For manually measured anthropometric labels, the measurement tools, measurement posture, number of measurers, number of repeat measurements, and measurement consistency should be specified to assess the impact of labeling errors on model training and testing results. Fifth are access and licensing specifications, including methods for public download or requesting access, data usage agreements, restrictions on commercial use, ethical statements, and recommended citation formats. Only when this information is fully disclosed can the dataset truly become a comparable, reproducible, and scalable benchmark resource.

### 5.3. Insufficient Validation of Cross-Ranch Generalization Ability

For visual body measurement systems for beef cattle, high accuracy of a model on a single ranch or a single dataset does not necessarily indicate its practical generalization ability [17]. There are significant differences among real-world ranches in terms of barn structure, ground surface materials, lighting conditions, camera mounting methods, animal breeds, coat color and texture, husbandry practices, background interference, and animal behavior patterns. If both training and testing data are sourced from the same farm, the model may learn features specific to that background, camera angle, or breed rather than stable phenotypic characteristics of animal body measurements. Therefore, cross-farm generalization ability should be a key metric for evaluating whether a visual body measurement system has practical application value.

Regarding validation methods, random image splitting alone is not recommended. Random splitting may result in highly similar samples-such as the same cow, adjacent frames within the same video sequence, or samples collected during the same time period-appearing simultaneously in both the training and test sets, leading to data leakage and overestimation of performance. For body measurement tasks, a more reasonable data split should treat individual animals, farms, time, breeds, and sensors as independent units. First, an “animal-independent split” should be adopted to ensure that the same cow does not appear in both the training and test sets. This strategy tests whether the model can generalize from seen individuals to unseen ones, rather than simply memorizing the appearance features of individual animals. Second, for datasets spanning multiple farms, a farm-independent split or “leave-one-farm-out” validation can be employed. This involves designating one farm at a time as a completely independent external test set, while using the remaining farms for training and validation, and repeating this process until every farm has served as an external test set. This method directly evaluates the model’s robustness in unfamiliar farm environments, including the effects of different barn structures, lighting conditions, floor materials, background interference, camera mounting methods, animal breeds, and husbandry practices.

In addition to farm-level splitting, more granular generalization validation should be configured based on the research objectives. For continuous video or high-frequency data, a time-independent split should be used to prevent adjacent frames or data from the same collection day from being included in both the training and test sets simultaneously. For datasets containing multiple breeds, a breed-independent split can be implemented that trains the model on a subset of breeds and tests it on breeds not used in training to evaluate the model’s adaptability to differences in body conformation, coat color and texture, and growth rates. For multi-sensor systems, a sensor-independent split can be implemented; for example, training the model using data from a specific RGB-D camera, a specific viewing angle, or a specific mounting height, and then testing it on another device or under different mounting conditions to evaluate the model’s robustness to device and calibration variations [39].

Regarding evaluation metrics, in addition to conventional regression metrics such as MAE, RMSE, MAPE, and R^2^, the performance difference between internal and external testing should also be reported. The cross-farm performance degradation rate can be used to quantify the loss in accuracy when the model is tested on an external farm after being tested in a similar scenario:

Cross-farm performance degradation rat = (External farm test error − Internal test error)/Internal test error × 100%

If a model exhibits low error in internal testing but shows a significant increase in error during external farm testing, this indicates that the model may be overfitted to specific farm conditions. Conversely, if the model maintains low error and minimal performance degradation in “leave-one-farm-out” validation, this provides stronger evidence of its suitability for real-world farm deployment. Therefore, future studies reporting body measurement accuracy should clearly specify whether the training, validation, and test sets were stratified by individual, farm, time, breed, or sensor, and should prioritize providing external farm test results. This will advance model evaluation from “accuracy comparisons across single datasets” to “verification of reliability in real-world applications”.

### 5.4. Vision-Based Body Measurement for Livestock Digital Twins and Predictive Management

As public datasets, standardized annotations, and cross-farm validation are gradually refined, vision-based body measurement can evolve from a tool for extracting individual body measurements into the foundational phenotypic layer of a livestock digital twin system [115,116,117]. The core of a livestock digital twin is not simply the creation of a three-dimensional animal model, but rather the continuous updating in a virtual space of the dynamic state corresponding to the real animal, including information on morphology, growth, body condition, health, behavior, nutrition, and environmental responses. For beef cattle management, visual phenotypic variables such as height, body length, chest width, chest depth, rump width, chest circumference, abdominal circumference, live weight estimation, and body condition score reflect an individual’s skeletal development, muscle deposition, nutritional status, and production potential, and serve as a crucial foundation for constructing individual digital twin models [118,119,120,121,122].

From a data flow perspective, visual body measurements can provide three key types of input for livestock digital twins. The first type is geometric morphological input, including two-dimensional contours, anatomical landmarks, depth maps, and three-dimensional point clouds. This data is used to describe the animal’s current body structure and serves as the basis for updating individual morphological models in digital twins [123]. The second category consists of time-series data, namely, body measurements, body condition scores, and body weight estimates obtained continuously across different growth stages. Compared to single measurements, continuous body measurement data can generate individual growth curves and body condition change trajectories, thereby identifying issues such as growth retardation, malnutrition, abnormal emaciation, or declining production performance. The third category is multi-source fusion input, which involves correlating visual data with RFID data or visual re-identification, automatic weighing, feed and water intake, activity levels, environmental temperature and humidity, and health records. Only by achieving synchronized mapping of individual identity, morphological status, behavioral status, and environmental status can a digital twin system transition from “static 3D reconstruction” to “dynamic individual modeling”.

Sensor fusion is a critical component in the construction of digital twins. Two-dimensional RGB images are low-cost and easy to deploy, making them suitable for large-scale continuous monitoring, but they lack depth information; RGB-D cameras can simultaneously capture color and depth data, making them suitable for semi-controlled passageway or pen environments; LiDAR can provide high-precision 3D point clouds and offers advantages in complex lighting conditions and open pasture environments, but it involves higher costs and greater data processing complexity; multi-view systems can mitigate occlusion issues associated with single-view systems but rely on sensor calibration, time synchronization, and point cloud registration [39]. Therefore, within the digital twin framework, different sensors should not be viewed simply as alternatives but rather as complementary data sources that collectively support individual state modeling. For example, RGB images can be used for object detection and individual identification; RGB-D or LiDAR can be used for 3D body surface reconstruction; automatic weighing data can be used to calibrate live weight prediction models; and feeding and environmental sensors can help explain the causes of body condition changes.

At the AI-driven predictive management level, the value of visual body measurement data lies not only in recording current status but also in predicting future changes. Continuous body measurements, weight estimates, body condition scores, feeding behavior, activity levels, environmental temperature and humidity, and health records can be collectively fed into machine learning or deep learning models to build models for growth prediction, precision nutrition, health early warning, and market-ready decision-making [120]. For example, if a cow’s growth rate is significantly below the herd average under identical feeding conditions, a digital twin system can further analyze feed intake, activity levels, and health records to determine whether the animal is experiencing inadequate nutrient intake, disease risk, or competitive disadvantage; if body condition scores continue to decline without significant changes in body weight, this may indicate abnormal energy metabolism or increased lactation or reproductive stress. Imamura et al. used 3D cameras to automatically evaluate beef cattle body condition scores [124], while Thi Thi Zin et al. further estimated body condition scores based on 3D cameras and regression analysis; such studies provide direct references for incorporating visual phenotypic data into health prediction, precision nutrition, and digital twin management [125].

Therefore, future research on vision-based beef cattle body measurement should shift from measuring individual body parameters toward a closed-loop framework encompassing real-time sensing, dynamic modeling, predictive analysis, and management decision-making. Within this framework, standardized public datasets provide the foundation for model training and objective performance comparison; cross-farm generalization validation ensures that algorithms can adapt to real-world production environments; and vision-based body measurement serves as a source of continuous, non-contact, and quantifiable phenotypic data for livestock digital twins. Only through the coordinated advancement of these three elements can vision-based body measurement technology truly support real-time phenotypic analysis, precision nutrition, health prediction, and production decision-making within intelligent livestock systems.

## 6. Conclusions and Outlook

### 6.1. Conclusions

Vision-based beef cattle body measurement technology provides an important means for non-contact, automated, and continuous phenotypic data collection in precision livestock farming. This paper systematically reviews major technical approaches, including 2D RGB image measurement, RGB-D depth measurement, LiDAR point cloud measurement, and multi-view fusion measurement, and summarizes key algorithms such as object detection, image segmentation, keypoint localization, 3D reconstruction, point cloud processing, and geometric calculations. Overall, 2D image-based methods offer the advantages of low cost, flexible deployment, and good real-time performance; however, their ability to represent 3D body measurement parameters-such as chest width, chest depth, chest circumference, and abdominal circumference-is limited. RGB-D methods can integrate both color and depth information, making them suitable for real-time measurements in semi-controlled ranch environments; however, they are susceptible to the effects of lighting, ranging distance, and depth noise. LiDAR and multi-view fusion methods can provide a more complete three-dimensional spatial structure and hold significant potential for complex environments and three-dimensional body modeling, but they also face challenges such as equipment costs, data processing complexity, sensor calibration, and system maintenance.

Existing research has demonstrated that visual body measurement holds great promise for tasks such as estimating height, length, width, and body weight, as well as body condition scoring; however, this field still faces three key limitations. First, public datasets and standardized annotation systems remain inadequate. Although some visual datasets for beef cattle are available for body condition scoring, body weight estimation, pose estimation, and individual identification, there are still few public benchmark datasets specifically designed for body measurement that include multimodal sensor data, standardized body measurement labels, individual metadata, and data collected across different pastures. Second, the generalization ability of models has not been sufficiently validated. Many studies still rely on performance evaluations conducted at a single farm or using random image splits, making it difficult to accurately reflect the models’ actual robustness across different farms, breeds, equipment, and temporal conditions. Third, the integration of visual body measurement data with intelligent livestock management systems still needs to be strengthened. Future research should not only focus on the automatic extraction of individual body measurement parameters but also on how continuous phenotypic data can be incorporated into digital twin models, health prediction models, and precision nutrition decision-making systems.

Therefore, vision-based beef cattle body measurement technology should evolve along the path of “standardized datasets-rigorous generalization validation-multi-source sensor fusion digital twins and predictive management”. By establishing public datasets that are multi-farm, multi-breed, multi-modal, and standard-annotated; adopting external validation strategies such as “leave-one-farm-out” validation; and integrating body measurements, body condition, body weight, behavioral, and environmental information into dynamic individual models, visual measurement technology is expected to evolve from a one-time measurement tool into a core phenotypic sensing module within intelligent livestock management systems. This approach will not only help improve the accuracy of beef cattle growth assessment, health monitoring, and production decision-making but will also provide critical support for the digitization, intelligent transformation, and sustainable development of precision livestock farming.

### 6.2. Outlook

Future research on vision-based beef cattle body measurement will gradually shift from the optimization of individual algorithms toward the coordinated development of data, models, systems, and applications. First, efforts should be strengthened to build standardized public datasets tailored to body measurement tasks. Existing beef cattle vision datasets are mostly focused on body condition scoring, body weight estimation, pose estimation, or individual identification; public datasets that specifically include standardized body measurement labels, multimodal sensor data, individual metadata, and data collected across different pastures remain limited. Therefore, future datasets should cover multiple farms, breeds, growth stages, and sensor conditions, and systematically provide RGB, RGB-D, LiDAR, multi-view images, manual body measurements, body weight, BCS, keypoints, segmentation masks, individual identifiers, and data collection environment information. At the same time, datasets should clearly define access methods, licensing agreements, and standard segmentation schemes to support fair comparisons among different methods.

Second, model evaluation should shift from random image splitting to rigorous cross-scenario generalization validation. For visual body measurement tasks, random splitting can easily result in the same cow or adjacent video frames appearing in both the training and test sets, thereby overestimating model performance. Future research should prioritize animal-independent, farm-independent, time-independent, breed-independent, and sensor-independent splits, and adopt “leave-one-farm-out” validation as a key protocol for evaluating cross-farm generalization capabilities. In addition to conventional error metrics, test errors from external farms and cross-farm performance degradation rates should be reported to accurately reflect the model’s deployment potential in different farm environments.

Third, efforts should be made to advance visual body measurement from monomodal perception toward multi-source sensor fusion. Two-dimensional RGB images, RGB-D depth maps, LiDAR point clouds, and multi-view images each have distinct characteristics in terms of cost, accuracy, robustness, and deployment complexity. Future systems should fuse visual data with RFID, automatic weighing, feed and water intake, activity level, and environmental sensor data based on different farm scenarios to achieve simultaneous perception of individual identity, morphology, behavior, environment, and production status. At the same time, lightweight deep learning models, edge computing, and on-device deployment remain critical technological pillars for enabling real-time monitoring [126].

Fourth, visual body measurement should further support livestock digital twins and AI-driven predictive management. Continuously collected body measurements, weight estimates, body condition scores, and 3D morphological data can form the phenotypic foundation of individual animal digital twins and, together with feeding, activity, environmental, and health records, construct dynamic individual models. Future research should focus more on the correlations between body measurement trends, body condition dynamics, health risks, and production performance, rather than merely on errors in single measurements. By incorporating visual phenotypic data into models for growth prediction, precision nutrition, disease early warning, and market-ready decision-making, vision-based body measurement technology is expected to evolve from a data collection tool into a core decision-support module within intelligent livestock management systems.

Finally, future research should also strengthen standardized evaluation and on-farm validation. Different studies should strive to report consistent sensor configurations, calibration methods, manual measurement protocols, data segmentation strategies, and evaluation metrics, and conduct external validation across multiple farms, breeds, and long-term continuous data sets. Only by establishing a unified framework for data standards, generalization validation, and closed-loop application can visual body measurement technology truly transition from laboratory or single-farm trials to large-scale, replicable, and scalable smart livestock applications.

## Figures and Tables

**Table 1:** Representative Studies on 2D Vision Technology in Body Measurement and Related Applications Over the Past Five Years.

Imaging Configuration	Sample Size	Measurement Indicators	Technical Process	Verification Method	Performance Indicators	References
Side view image of a standard RGB camera	Images of 30 beef cattle	Body diagonal length, body height, chest depth, hoof circumference	YOLOv5s detection, Lite-HRNet keypoint localization, monocular depth estimation, geometric conversion	Manual measurement comparison	The average relative errors were 6.75%, 7.55%, 8.00%, and 8.97%, respectively	[37]
Mobile/Regular RGB Photo	108 Korqin Yellow Cattle	Live weight	Keypoint extraction, automatic body surface measurement, deep regression	Comparison Experiments Between Public Datasets and Self-Built Datasets	The weight estimation accuracy is greater than 97%	[23]
Two-dimensional imaging system	Training set: 34,150 manual BCS annotations; Test set: 9657 single BCS records	BCS	2D imaging, machine learning, ordinal regression	Comparison with manual scoring, observer consistency, and backfat thickness	Kw = 0.69; agreement rate within ±0.25 error range is 84.6%; agreement rate within ±0.50 error range is 94.8%	[38]
RGB image, depth image, RGB-depth fusion, segmented image, segmented-depth fusion	Self-built dataset	Live weight	Three deep learning baseline models; Vision Transformer zero-shot segmentation	Multimodal contrast experiment	Comparison of the effects of different modalities using four indicators: MAE, RMSE, MAPE, and R^2^	[39]

Note: MAE represents Mean Absolute Error; RMSE represents Root Mean Square Error; MAPE represents Mean Absolute Percentage Error; kw = Weighted Kappa coefficient.

**Table 2:** Comprehensive Comparison of 2D Image, RGB-D, and LiDAR Body Measurement Methods.

Comparison Indicator	2D Image Method	RGB-D Method	LiDAR Method
Data Source	RGB Images	RGB Images and Depth Maps	3D Point Clouds
Hardware Costs	Low	Moderate	High
Spatial Info Acquisition Capability	Mainly reflects 2D contours, textures, and visible key point information	Can synchronously acquire color and depth information	Can acquire high-precision 3D spatial structural information
Suitable Measurement Indicators	Height, body length, and other visible contour parameters	Height, body length, body width, body depth, and partial dimension parameters	Height, body length, body width, body depth, and 3D body shape parameters
Measurement Accuracy	Medium; significantly affected by scale calibration, posture, and shooting angle	Relatively high; affected by depth noise, ranging limits, and occlusion	Usually high; affected by point cloud density, occlusion, and registration quality
Environmental Adaptability	Sensitive to lighting changes, background interference, and occlusion	Good applicability in semi-controlled environments, but limited under strong light and reflective conditions	Insensitive to lighting changes, suitable for open or complex outdoor environments
Data Processing Complexity	Low	Moderate	High
Main Advantages	Low cost, simple deployment	Balances cost, real-time performance, and spatial acquisition capability	Strong 3D representation capability, environmental robustness
Main Limitations	Lacks depth information, 3D body dimension estimation is limited	Depth maps are susceptible to noise, lighting, and effective ranging limits	High equipment cost, point cloud processing and system integration are complex

**Table 3:** Representative Research on Multi-View Fusion Systems in Recent Years.

Number and Types of Sensors	Fusion Method	Registration Accuracy	Application Scenario	References
3 RGB-D cameras	Non-rigid 3D shape reconstruction	Measurement error < 3%	Non-contact automatic measurement of livestock body size	[52]
LiDAR camera	Multi-frame Weighted Edge Feature Alignment	Translation accuracy 0.977 cm, rotation accuracy 0.086°	Automatic Calibration of LiDAR-Camera Extrinsic Parameters	[53]
3 RGB-D cameras	Coarse and Fine Registration Fusion of Multi-View Point Clouds	Average relative error: body height 2.32%, body length 2.27%, abdominal circumference 3.67%, chest circumference 5.22%	Outdoor full-body automatic measurement of beef cattle	[24]
Multiple depth cameras	2D Keypoint Detection 3D Point Cloud Projection	Measure MAPE < 10%	2D and 3D Integrated Measurement of Livestock Body Size	[25]

**Table 4:** Performance Comparison of 3D Point Cloud Segmentation Methods.

Method Category	Representative Algorithm	Advantages	Disadvantages	Computational Efficiency	References
Geometric Rules Method	DBSCAN Surface Segmentation Based on Quadratic Fitting	The DBSCAN method based on quadratic fitting enhances noise robustness through normal correction and density-reachable condition expansion, with surface extraction accuracy superior to RANSAC, RG, LCCP, and other methods.	The DBSCAN method based on quadratic fitting is quite sensitive to normal estimation errors, and the merging criterion relies on empirical thresholds.	Medium	[68]
Traditional feature learning methods	Normal vector, curvature, multi-scale local geometric features classifier	Relatively strong interpretability, with certain adaptability to small sample scenarios	Relying on feature engineering, cross-scenario generalization capability is limited	Medium	[69]
Deep Learning	Point Transformer	Strong capability in global relationship modeling, able to effectively capture long-range dependencies	The model is relatively complex, and the cost of training and deployment is high.	Medium	[70]
Deep Learning	Point Transformer V2	Enhance the ability to fuse local and global features, achieving better performance in large-scale point cloud segmentation	The network structure is more complex and requires higher computing power.	Medium	[71]
Deep Learning	PointNeXt	Achieve a good balance between accuracy, efficiency, and scalability	Still requires support from a relatively large scale of training data	High	[72]
Deep Learning	Point-BERT	Enhance feature representation and generalization ability through self-supervised pre-training	The pre-training and fine-tuning process is relatively complex.	Medium	[73]
Deep Learning	MLGCN	Strong capability in graph-structured modeling, balancing accuracy and lightweight design	Sensitive to neighborhood composition and graph computation process	Medium	[74]

**Table 5:** Comparison of Livestock Keypoint Detection Methods Based on Deep Learning in the Past Five Years.

Method Type	Data Modality	Representative Algorithm	Core Idea	Advantage	Limitation	References
2D keypoint detection	RGB image	SimCC, SAR, YOLOX-Pose	SimCC, SAR, and YOLOX-Pose frameworks combined with various backbone networks are used for comprehensive benchmark evaluation, assessing keypoint detection accuracy, robustness, and computational efficiency.	High precision, stable positioning	Can only obtain two-dimensional coordinates	[84]
2D keypoint detection	RGB image	RTMPose	Lightweight pose estimation network for real-time keypoint detection	Fast reasoning speed	Sensitive to blockage	[85]
2D keypoint detection	RGB image	Lightweight cattle pose network	Introducing attention mechanisms and reparameterization structures	The model is lightweight and easy to deploy	Data-dependent	[86]
2D and 3D integration	2D image	DeepLabCut	Using the RealSense D455 depth camera to collect data, employing DeepLabCut for keypoint detection, and combining it with a dynamic depth replacement optimization algorithm to calculate body measurement parameters	Meet the real-time and efficient measurement needs of large-scale farming	Relying on camera calibration, measurement accuracy is affected by the quality of depth images	[87]
Key Points and Point Cloud Reconstruction	RGB-D	YOLOv8-Pose-LSCDH	After predicting key points, map them to the 3D point cloud	Low measurement error	Dependence on depth quality	[28]
3D Point Cloud Keypoint Detection	RGB-D	YOLOv8-DMC	Integrates the DRAMiTransformer, MHSA-C2f, and CASimAM attention modules to enhance feature extraction capabilities, combined with deep completion and point cloud filtering to achieve 3D keypoint localization	Support edge device deployment	Depends on depth quality, only suitable for side-view image capture scenarios	[88]

**Table 6:** Comparison of Methods for Calculating Visual Body Size.

Method Type	Data Input	Core Idea	Advantages	Disadvantage	References
Geometric Model	Unilateral point cloud	Repair point cloud holes through moving least squares, using polynomial fitting and cubic B-spline fitting to calculate body height, chest circumference, and body length	Easy to implement, low cost for single-sided collection devices, and after repair, the accuracy can meet basic measurement requirements.	Easily affected by posture and occlusion, sensitive to the completeness of point clouds, with noticeable deviation in fitted curves where curvature changes significantly	[104]
Three-dimensional geometry method	Point cloud	UAV-mounted LiDAR scans acquire point clouds, use a dual-rotation algorithm for attitude normalization, and establish a linear regression model to estimate hip height	Provides real spatial information, can be collected non-contact under natural breeding conditions, with a large measurement range	Sensitive to flight altitude and speed, requiring the removal of abnormal data; high equipment cost; strong dependence on point cloud quality	[48]
Key point method	Point cloud	① DUOS-PointNet semantic segmentation divides the bull body into seven parts, calculating body measurements by combining point cloud slicing and density measurement; ② YOLOv8-DMC detects key points, achieving 2D-3D mapping by generating color point clouds through depth completion and bilateral filtering	High degree of automation, capable of handling complex structures, high measurement accuracy, supports edge device deployment	Depends on the accuracy of the segmentation model and the amount of training data; keypoint detection robustness decreases under occlusion; computational complexity is higher than geometric models.	[88,105]

## Data Availability

No new data were created or analyzed in this study. Data sharing is not applicable to this article.
